# Global burden, disparities, and determinants of postpartum haemorrhage among women who gave birth: an umbrella review of systematic reviews and meta-analyses

**DOI:** 10.3389/frph.2025.1721550

**Published:** 2025-12-18

**Authors:** Dereje Bayissa Demissie, Doreen Kainyu Kaura, Stefan Gebhardt

**Affiliations:** 1 Faculty of Medicine and Health Sciences, Stellenbosch University, Cape Town, South Africa; 2 School of Nursing, Faculty of Community Health Sciences, University of the Western Cape, Cape Town, South Africa; 3 Head: Clinical Department Obstetrics and Gynaecology, Faculty of Medicine and Health Sciences, Stellenbosch University, Cape Town, South Africa

**Keywords:** global prevalence, health disparities, maternal health, postpartum haemorrhage (PPH), risk factors, systematic reviews, umbrella review

## Abstract

**Background:**

Postpartum haemorrhage (PPH) is a preventable yet leading cause of maternal death, demanding urgent attention and action. Every year, tens of thousands of women die from postpartum haemorrhage a tragedy that is both preventable and unacceptable. Each maternal death represents a systemic failure. This umbrella review consolidates global evidence on burden, disparities, and determinants of postpartum haemorrhage (PPH) to inform policymaker's evidence decision making.

**Methods:**

A comprehensive literature search was conducted between July 30 and August 15, 2025, across PubMed, Scopus, Science Direct, Web of Science, Cochrane, and related review databases to identify systematic reviews and meta-analyses on the prevalence, disparities, and determinants of postpartum haemorrhage among women who gave birth. Study quality was assessed using the AMSTAR tool. Heterogeneity was examined with Cochran's Q and *I*², publication bias via Egger's test and funnel plots, and pooled effect sizes estimated through random-effects meta-analysis using Stata 19. The review protocol was registered in PROSPERO (CRD420251121022).

**Result:**

This umbrella review included 17 systematic reviews with sample size of over 21 million women to estimate the global pooled prevalence of postpartum haemorrhage (PPH) at 9.97% (95% CI: 6.90%–13.04%), with (*p* < 0.001). Based on studies using objective blood loss measurement (≥500 mL per 100 women giving birth), the prevalence increased to 11.25% (95% CI: 8.78%–13.72%) with *p* < 0.001.

Globally pooled prevalence of severe postpartum haemorrhage is estimated at 4.52% (95% CI: 2.47%–6.57%) with *p* < 0.001, indicating that nearly 1 in 20 women experience life-threatening bleeding after childbirth.

This study identified key risk factors for postpartum haemorrhage, including maternal age, lack of antenatal care, obstetric complications, and rural residence. These determinants highlight the need for targeted prevention strategies to reduce PPH-related morbidity and improve maternal health outcomes.

**Conclusion and recommendation:**

The study underscores postpartum haemorrhage (PPH) as a critical global health issue, with approximately 1 in 10 women experiencing PPH worldwide and 1 in 20 affected by severe PPH, highlighting the urgent need to address persistent disparities. An immediate policy action is essential to guarantee timely, effective care and to uphold maternal health as a fundamental human right. As the global call reminds us: “No woman should die giving life.”

**Systematic Review Registration:**

[https://www.crd.york.ac.uk/PROSPERO/view/CRD420251121022], identifier CRD420251121022.

## Background

Postpartum haemorrhage (PPH) is commonly defined as a blood loss of 500 mL or more within 24 h after childbirth. It remains the leading cause of maternal mortality worldwide, affecting approximately 14 million women annually and resulting in around 70,000 maternal deaths (1). Severe postpartum haemorrhage (SPPH) is defined as blood loss from the genital tract of 1,000 mL or more in the first 24 h after the delivery of the baby (2, 3).

Postpartum haemorrhage (PPH) accounts for approximately 27% of maternal deaths globally, affecting 5%–10% of all deliveries. The leading cause of PPH is uterine atony, responsible for nearly 70% of cases. While high-income countries have significantly reduced PPH-related mortality through timely and effective interventions, low- and middle-income countries continue to bear the highest burden. This concerning trend is underscored by World Health Organization (WHO) data, which identifies PPH as the leading cause of maternal mortality worldwide, despite being both preventable and treatable (4). In response to this persistent challenge, the World Health Organization, in collaboration with key stakeholders, has developed a comprehensive Roadmap (2023–2030) to combat PPH. This strategic framework outlines targeted goals, activities, and milestones across research, normative guidance, implementation, and advocacy, with a strong focus on countries bearing the highest burden (5). The Roadmap calls for urgent investment in health system strengthening and the adoption of standardized, evidence-based interventions to accelerate progress toward SDG target 3.1 and ensure safer childbirth for all women (5).

Over 80% of postpartum haemorrhage (PPH) deaths occur in sub-Saharan Africa and South Asia. In sub-Saharan Africa, PPH prevalence is 8.6%, influenced by maternal age, parity, and rural residence. LMICs face maternal mortality rates up to 100 per 100,000 live births (6). PPH is largely preventable through active labour management, uterotonics like oxytocin and misoprostol, and timely use of tranexamic acid. WHO's 2023–2030 PPH Roadmap targets LMICs, aligning with Sustainable Development Goal 3.1 to reduce maternal mortality (5).

Previous systematic review and meta-analysis of postpartum haemorrhage (PPH) in sub-Saharan Africa found that the magnitude of PPH was considerably higher than in other regions (7) and in Ethiopia reveals a high prevalence, with estimates ranging from 11.14% to 12.88% in Ethiopia (8). Some previous systematic review and meta-analysis identified several risk factors for PPH were identified across studies, including lack of antenatal care follow-up, multiparty, previous history of PPH, advanced maternal age, and prolonged labour (7–9). Additional factors such as antepartum haemorrhage, twin pregnancy, and induction of labour were also associated with increased risk of PPH (7). The studies emphasize the importance of improving antenatal care attendance and closely monitoring women with risk factors to reduce the incidence of PPH in these regions (7, 8).

Globally, numerous meta-analyses, systematic reviews, and primary studies have examined postpartum haemorrhage (PPH) prevalence, magnitude, incidence, and pooled determinants or factors increases odds of postpartum haemorrhage across various contexts, including sub-regional, national, and local levels. However, inconsistent findings across these studies’ present challenges for healthcare programs and clinical decision-making. To address these discrepancies, it is essential to consolidate evidence to the extent and contributing factors of postpartum haemorrhage (PPH). Therefore, the primary objective of this umbrella review is to provide a comprehensive synthesis of combined estimates on postpartum haemorrhage prevalence or magnitude, incidence, and pooled determinants or factors increases odds of postpartum haemorrhage across various contexts, including globally, sub-regional, national, and local levels to inform and strengthen health policy, service delivery, and maternal health equity worldwide.

General objective: The aim of this umbrella review was to examine the Global Burden, Disparities, and Determinants of Postpartum Haemorrhage: An Umbrella Review of Systematic Reviews and Meta-Analyses.

## Review objectives

To describe global patterns and Trends of postpartum haemorrhage (PPH) including incidence, prevalence, and outcomes using evidence from systematic reviews and meta-analyses.

To estimate global pooled prevalence postpartum haemorrhage.

To compare regional disparities in burden of postpartum haemorrhage (PPH) across continents.

To identify key pooled determinants postpartum haemorrhage among women who gave birth.

To assess the methodological quality and consistency of current systematic reviews and meta-analyses on postpartum haemorrhage, with the aim of informing future research priorities and guiding evidence-based healthcare policies.

To informing maternal health policy through aggregated global evidence.

## Methods

This umbrella review was conducted following established methodologies for synthesizing multiple systematic reviews (10). The procedure adheres to the umbrella review methodology developed by the Joanna Briggs Institute (11). It involved a systematic synthesis of eligible systematic reviews and meta-analyses (SRMs) that summarize the global prevalence, determinants, postpartum haemorrhage among women who gave birth *n*.

This umbrella review was conducted in accordance with the Preferred Reporting Items for Systematic Reviews and Meta-Analysis (PRISMA) and was registered in PROSPERO with reference number of CRD420251121022, published on 12August 2025, available from https://www.crd.york.ac.uk/PROSPERO/view/CRD420251121022.

## Search strategy

Five international online databases PubMed, Scopus, Science Direct, Web of Science, and databases specific to systematic reviews and meta-analysis such as the Cochrane database of systematic reviews and the database of abstracts of reviews of effects were searched for systematic reviews and meta-analyses (SRMs) on the prevalence, disparities, and determinants of postpartum haemorrhage among women who gave birth. A comprehensive search strategy was employed using adapted PICO questions, developed from relevant keywords and Medical Subject Headings (MeSH). These terms were combined using Boolean operators “OR” and “AND” to ensure thorough and inclusive search.

PICO stands for Patient/Population, Intervention, Comparison, and Outcome.

a) Population: Female (adolescents, youths,) and women of reproductive age.
b) Outcome: postpartum haemorrhage (PPH) prevalence, magnitude, incidence, determinants, predictors, associated factors, correlates, risk factors of postpartum haemorrhage on maternal health.
c) Study design: systematic review, meta-analysis of observational studies
d) Setting (context): Globally

Both published and unpublished studies were included in this umbrella review.

A comprehensive literature search was conducted from July 30, 2025, to August 15, 2025, covering studies published from January 2000 to July 1, 2025. Two independent researchers carried out the search, and any discrepancies were resolved through discussion and consensus with the remaining authors. A sample search strategy for PubMed was developed using a combination of Medical Subject Headings (MeSH) terms and free-text keywords. The key terms to retrieve systematic review and meta-analysis studies (magnitude OR prevalence AND postpartum haemorrhage OR bleeding AND globally. The sample search strategy used to identify relevant articles using keywords for PubMed Database search was ((((((post-partum haemorrhage OR postpartum bleeding, OR maternal bleeding OR “PPH”, OR “magnitude of PPH” OR Prevalence of PPH OR Epidemiology) AND (determinants OR associated factors OR predictors) OR “postpartum women”, OR “maternal health care” OR AND (systematic review and meta-analysis)))) in details are provided in Supplementary File 1.

## Eligibility criteria

### Inclusion criteria

This umbrella review included all available systematic reviews and meta-analyses (SRMs) that met predefined criteria: a clearly defined literature search strategy, quality appraisal of included studies using appropriate tools, and a standardized approach to pooling data and summarizing estimates for women of reproductive age. Both published and unpublished studies were considered, with language restricted to English. A comprehensive literature search was conducted between July 30, 2025, to August 15, 2025, covering studies published from January 2000 to July 1, 2025. This broad inclusion aimed to capture comprehensive evidence on the burden, determinants, and disparities of PPH across different settings.

### Exclusion criteria

Studies were excluded if they did not report on the prevalence, magnitude, incidence, determinants, predictors, associated factors, correlates, risk factors, or adverse effects of postpartum haemorrhage. Qualitative reviews, narrative reviews, editorials, correspondence, abstracts, and methodological papers were also excluded. Additionally, literature reviews lacking a clearly defined research question, search strategy, or article selection process were not considered. This rigorous screening ensured the inclusion of high-quality, relevant evidence to support a comprehensive synthesis of PPH.

### Data extraction

Data from the included SRM studies were extracted using a standardized Excel form. The extracted information was included study identification, review aim postpartum haemorrhage (PPH) prevalence, magnitude, incidence, determinants, predictors, associated factors, correlates, risk factors, adverse effects of postpartum haemorrhage on maternal health. Additional data were covered effect sizes (OR/RR with 95% CI), the number and design of primary studies, sample sizes, methods and scores for publication bias and quality assessment, data synthesis models, and the main conclusions. Full extraction details are provided in Supplementary File 1.

### Risk of bias assessment

All the included studies were critically appraised to assess the validity and scoring of the results. This Umbrella review was used the Assessment of Multiple Systematic Reviews (AMSTAR−2) tool to ensure the methodological and evidence quality of the included SRM studies (10, 12) in in details are provided in Supplementary File 2.

#### Management of overlapping studies

We addressed overlapping primary studies by creating a citation matrix to identify duplicates and calculating the Corrected Covered Area (CCA) to quantify overlap. When overlap was minimal, all reviews were retained and synthesized narratively. For substantial overlap, we prioritized the most comprehensive and methodologically robust review based on AMSTAR-2 scores, using others for context. The process was documented in the methods section, with a summary table provided in Supplementary Materials to ensure transparency and prevent bias in pooled estimates.

### Data synthesis

Both narrative (qualitative) and quantitative approaches were used to summarize the findings of the included SRM studies. When multiple estimates are reported for prevalence postpartum haemorrhage associated factors, among women who gave birth, the range of estimates were presented, and a pooled summary estimate was calculated. The choice of meta-analysis model was guided by the level of heterogeneity, assessed using Higgins’ *I*² statistic (13). Due to anticipated high between-study heterogeneity, the DerSimonian-Laird random-effects model was employed (13).

Publication bias was assessed if at least 10 studies are included, as this is the minimum typically required for such evaluation (10, 14). Meta-analysis was involved calculating pooled estimates of prevalence postpartum haemorrhage directly reported from the included studies. Heterogeneity was assessed using both Cochran's *Q* test and the *I*² statistic (15). An *I*² value greater than 50% and a Cochran's *Q* test *p*-value < 0.05 was indicate significant heterogeneity, warranting the use of a random-effects model (15).

Pooled estimates were calculated using STATA version 17, employing the “metaprop” command with sample size as the weighting variable and 95% confidence intervals. To explore sources of heterogeneity, we conducted subgroup analyses by year of publication and continent, and performed meta-regression to examine potential moderators. This approach ensured robust pooled estimates and improved interpretability. Quantitative analyses were conducted in STATA version 17.0. A summary of determinants of postpartum haemorrhage, along with their respective adjusted odds ratios was computed.

### Ethical consideration

This study does not require ethical approval or informed consent from participants, as it is based solely on data extracted from previously published SRM studies.

## Results

### Study screening and selection

A total of 261 systematic reviews and meta-analyses were initially identified through database searches, as detailed in Supplementary File S2: Search Strategy. Two reviewers independently screened titles and abstracts, identifying 29 potentially eligible articles for full-text review. An additional 8 articles were found through reference checks and supplementary searches, resulting in a total of 37 full-text articles assessed for eligibility (3, 6–9, 16–46). Of these, 17 met the predefined inclusion criteria and were included in this umbrella review of systematic reviews and meta-analyses with total sample size of 21,299,318 women of reproductive age who experienced postpartum haemorrhage (PPH) (3, 7–9, 34–46). Of the 37 full-text articles assessed for eligibility, 20 were excluded based on predefined criteria to ensure methodological rigor and relevance. The most common reasons for exclusion were: Studies reporting outcomes that did not meet the inclusion criteria (unrelated to postpartum haemorrhage prevalence or associated factors or determinants), and articles classified as study protocols without primary data or results by ensuring that only studies meeting the eligibility criteria were synthesized.

**Figure 1:**
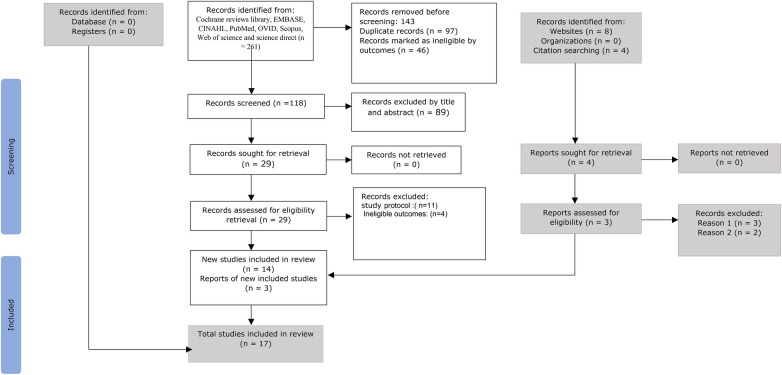
PRISMA 2020 flow diagram for new systematic reviews, which included searches of databases, registers, and other sources (47).

The study selection process, including the number of records identified, screened, assessed for eligibility, and included, is illustrated in the PRISMA flow diagram (Figure 1).

### Highlights of authors’ conclusions postpartum haemorrhage (PPH) studies

This umbrella review synthesizes findings from multiple systematic reviews and meta-analyses. The global pooled prevalence of PPH varies widely, with the highest rates reported in Sub-Saharan Africa and among specific clinical populations such as women with placenta previa and non-cirrhotic portal hypertension. However, the overall prevalence may be influenced by the number of studies included from high-burden regions such as Africa (7–9, 40, 41, 44).

Globally, PPH prevalence ranges from 3.5% to 22.3%, with significant variation due to differences in measurement methods, study settings, and population characteristics. Authors consistently emphasize the need for standardized blood loss measurement techniques, improved data collection, and targeted interventions. Risk factors such as older maternal age, multiparity, and lack of antenatal care, prolonged labor, and previous PPH history are frequently cited (3, 9, 34, 35, 38, 43).

Special populations including women with placenta previa, portal hypertension, and those delivering in low-resource settings face elevated risks. Authors call for enhanced obstetric care, early risk identification, and strengthened health system responses to reduce maternal morbidity and mortality (34). All included studies underwent rigorous quality assessment using the AMSTAR-2 tool, with scores consistently ranging from 11 to 16, ensuring methodological robustness and reliability of the findings. For further detailed information presented in Supplementary Table S1. Due to the large number of studies, summarizing all details in a single table was not feasible. Therefore, the tables were organized based on postpartum haemorrhage (PPH) outcome measurements. Table 1 presents highlights of authors’ conclusions on global PPH measured non-objectively. Table 2 shows disparities in the burden of PPH across continents. Table 3 summarizes authors’ conclusions from studies using objective blood loss measurement methods, and Table 4 provides global estimates of severe postpartum haemorrhage (SPPH) from included studies. Detailed information for each category is available in the Supplementary Files of table to 4.

**Table 1:** Summarizes the total number of included studies, study settings, number of countries represented, overall sample size, and key conclusions reported by the authors 2025.

Author (year)	Review aim	Search strategy	# No studies	Study setting	Sample size	PPH blood loss >=500 mL	Authors conclusion	Quality AMSTAR-2
Bestman, Pan, & Luo, (44)	To assess the prevalence and risk factors of PPH in Africa, so as to provide scientific evidence base findings that might be used to ensure maternal safety	Google scholar, PubMed, EMBASE, Scopus, Web of Sciences, and Grey literature databases	12	Africa	130,570	3.51	This systematic review found that 3.51% prevalence of postpartum haemorrhage (PPH) in Africa. Common risk factors spontaneous vaginal delivery, older maternal age, and multiparty were consistently reported but varied in strength across countries. The findings highlight the need for region-specific strategies and future research focused on PPH prevention to enhance maternal and child health.	**12**
Calvert et al., (3)	To provide regional estimates of the prevalence of maternal haemorrhage and explore the effect of methodological differences between studies on any observed regional variation	Google scholar, PubMed, EMBASE, Scopus, Web of Sciences, and Grey literature databases	63	Globally	1,003,694	10.8	Postpartum haemorrhage (PPH) prevalence varies globally, with Africa showing the highest rates and Asia the lowest. Measurement methods and regional differences significantly affect estimates. Visual estimation often underreports blood loss. Standardizing measurement techniques and improving data collection—especially in developing countries—is essential for accurate global estimates and better maternal health outcomes. Objective methods are strongly recommended.	13
Carroli, Cuesta, Abalos, & Gulmezoglu, (38)	This study conducted a systematic review to assess the global and regional prevalence of postpartum haemorrhage (PPH), aiming to understand its magnitude across diverse settings.	Google scholar, PubMed, EMBASE, Scopus, Web of Sciences, and Grey literature databases	120	Globally	3,815,034	6.09	This systematic review analyzed 120 datasets on postpartum haemorrhage (PPH) and 70 on severe PPH (SPPH), covering over 4.3 million women. PPH and SPPH prevalence were approximately 6% and 1.86%, respectively, with significant regional variation. Reliable global estimates require well-designed studies, standardized measurement methods, and a comprehensive global survey to understand PPH's true magnitude and impact.	12
Fan et al., (34)	The aim of this study was to calculate the average point incidence of PPH in women with placenta previa	Google scholar, PubMed, EMBASE, Scopus, Web of Sciences, and Grey literature databases	11	Globally	5,146	22.30	The summary estimate of the incidence of PPH among women with placenta previa was considerable in this systematic review. The results will be crucial in prevention, treatment, and identification of PPH among pregnant women with placenta previa and will be contributed to the planning and implantation of relevant public health strategies.	14
Giri, Sahoo, Sundaram, & Shukla, (43)	To determine the pooled prevalence of postpartum haemorrhage (PPH) among pregnant women diagnosed with non-cirrhotic portal hypertension (NCPH)	Google scholar, PubMed, EMBASE, Scopus, Web of Sciences, and Grey literature databases	10	Globally	337	4.70	A systematic review of 10 studies involving 337 pregnancies reported a pooled postpartum haemorrhage (PPH) rate of 4.7% among patients with non-cirrhotic portal hypertension (NCPH)	13
Huang et al., (35)	To estimate the incidence and identify risk factors of postpartum haemorrhage (PPH) after vaginal delivery	PubMed, Cochrane Library, CINAHL, Web of Science, EMBASE, and ClinicalTrials.gov databases were systematically searched	36	Globally	30,50,532	17.00	This review of 36 studies found PPH rates of 17% (≥500 mL) and 6% (≥1,000 mL), identifying 41 risk factors across five categories. Rising global PPH incidence highlights the need for obstetric providers to address labor-related risks—prolonged labor, oxytocin use, and genital trauma—to reduce maternal morbidity.	13
Moyo, Dzinamarira, Moyo, Murewanhema, & Ross, (7)	To provide an accurate estimation of the prevalence of PPH and to identify regional risk variables.	Google Scholar, EMBASE, Science Direct, CINAHL, MEDLINE, Africa Journals Online (AJOL), SCOPUS, and PubMed	26	Sub-Saharan Africa	106,640	8.60	Postpartum haemorrhage (PPH) is the leading cause of maternal death globally, with a significantly higher prevalence in Sub-Saharan Africa. It poses both immediate and long-term health risks. Despite established management strategies, individual risk factors are often unclear. This systematic review categorizes PPH risk factors into maternal, antenatal, intrapartum, and postpartum groups, highlighting the need for improved identification and prevention efforts.	14
Nigussie, Girma, Molla, Tamir, & Tilahun, (9)	To estimate the pooled magnitude of postpartum haemorrhage and the pooled effect size of the associated factors in Ethiopia	PubMed/MEDLINE online, Science Direct, Hinari, Cochrane Library, CINAHL, African Journals Online, Google and Google Scholars databases	21	Ethiopia	93,898	8.24	The pooled magnitude of postpartum haemorrhage among post-natal mothers in Ethiopia was moderately high. The finding of this study will strongly help different stakeholder working in maternal and child health to focus on the main contributors’ factors to reduce post-partum haemorrhage among postnatal mothers. Health professionals attending labor and delivery should give more attention to advanced aged mothers, grand-multipara mothers and mothers who had a history of post-partum haemorrhage due to higher risk for postpartum haemorrhagehaemorrhage. Encouraging to continue ANC visit and prevent prolonged labor should also be recommended to decrease postpartum haemorrhage.	15
Tiruneh, McLelland, & Plummer, (41)	To determine the pooled incidence of primary postpartum haemorrhage among women following in-hospital births.	searched electronic databases of Ovid MEDLINE, Ovid Emcare, Embase, PsycINFO, and CINAHL		Globally	7,062	12.00	The incidence of primary postpartum haemorrhage following in-hospital births was high, and suggest that preventive strategies implemented to reduce its occurrence needs further strengthening using training. Key messages: The result of this review suggests that globally at least one in ten women experience a primary postpartum haemorrhage following in-hospital births. This is higher than anticipated. The application of the recommended strategies for the prevention of primary postpartum haemorrhage should be re-emphasized.	11
Tolossa, Fetensa, Zewde, Besho, & Jidha, (8)	To estimates the pooled magnitude of PPH and factors associated with PPH among women who gave birth in Ethiopia	Electronic databases such as Medline, Pub Med, Cochrane library, the Web of Science, and Google Scholar were used to search for articles	9	Ethiopia	4,032	11.14	In Ethiopia the magnitude of PPH was high, and lack of ANC up follow-up, being multipara, and having a previous history of PPH were risk factors for postpartum haemorrhage. Thus, improving antenatal care follow-up is needed to decrease the magnitude of postpartum haemorrhage. All mothers need to be assessed carefully during the 24 h of post-delivery in the health institutions and health care providers need to strictly follow laboring mothers with partograph to detect and manage risk factors that cause PPH. In addition, all pregnant women need to receive health education and awareness to give birth in health institutions, and ANC follow up.	14
Yoseph Merkeb Alamneh, (40)	to assess the pooled prevalence and associated factors of postpartum haemorrhage among mothers at public health institutions in Ethiopia	MEDLINE/PubMed, EMBASE, Scopus, and Grey literature databases, Google Scholar, Science Direct and Cochrane library were extensively searched.	11	Ethiopia	16,416	12.5	A systematic review and meta-analysis found that postpartum haemorrhage (PPH) prevalence among mothers delivering at Ethiopia's public health institutions remains high, despite government prevention strategies. Key predictors include older age, rural residency, and grand multiparity, lack of antenatal care, previous PPH history, and delivery mode. Targeted interventions and increased vigilance during labor are essential to improve maternal outcomes and ensure preparedness for PPH management in all childbirth cases.	16
Total	319		82,33,361	Average 10.625				

**Table 2:** summary of included studies on postpartum haemorrhage (PPH) burden across continents 2000–2025.

Author (year)	Review aim	Search strategy	No of included studies	Study setting	Continent	Sample size	Blood loss >=500 mL	SE	Quality AMSTAR-2
Bestman, Pan, & Luo, (44)	To assess the prevalence and risk factors of PPH in Africa, so as to provide scientific evidence base findings that might be used to ensure maternal safety	Google scholar, PubMed, EMBASE, Scopus, Web of Sciences, and Grey literature databases	12	Africa	Africa	130,570	3.51	0.00,82,143	12
Calvert et al., (3)	To provide regional estimates of the prevalence of maternal haemorrhage and explore the effect of methodological differences between studies on any observed regional variation	Google scholar, PubMed, EMBASE, Scopus, Web of Sciences, and Grey literature databases	3	Africa	Africa	2,738	25.73	0.48,20,757	13
			6	Latin America and the Caribbean	Latin America and the Caribbean	23,129	8.2	0.05,05,237	
			7	Northern America	Northern America	28,580	13.1	0.07,44,727	
			20	Asia	Asia	215,611	8.5	0.01,71,951	
			18	Europe	Europe	378,617	12.7	0.01,98,105	
			9	Oceania	Oceania	355,019	7.2	0.01,12,134	
			2	Africa	Africa	1,889	5.1	0.105,211	
			4	Latin America and the Caribbean	Latin America and the Caribbean	15,551	3.3	0.02,20,923	
			4	Northern America	Northern America	21,744	4.3	0.02,55,459	
			11	Asia	Asia	11,416	1.9	0.01,22,389	
			18	Europe	Europe	452,116	2.8	0.00,33,388	
			1	Oceania	Oceania	330	3	0.13,484	
Carroli, Cuesta, Abalos, & Gulmezoglu, (38)	This study conducted a systematic review to assess the global and regional prevalence of postpartum haemorrhage (PPH), aiming to understand its magnitude across diverse settings.			Africa	Africa	14,443	10.45	0.08,26,885	12
				Eastern Africa	Africa	499	14.23	0.61,42,315	
				Middle Africa	Africa	2,410	18.67	0.36,99,831	
				Western Africa	Africa	11,534	8.57	0.07,49,978	
				Asia	Asia	391,141	2.55	0.00,31,788	
				Eastern Asia	Asia	186,749	3.96	0.00,79,225	
				South-Central Asia	Asia	8,659	4.35	0.04,10,235	
				South-Eastern Asia	Asia	3,835	4.88	0.07,02,657	
				Western Asia	Asia	191,898	1.05	0.00,05,231	
				Europe	Europe	32,95,864	6.38	0.00,32,271	
				Northern Europe	Europe	32,86,467	6.37	0.00,32,262	
				Western Europe	Europe	9,393	9.38	0.091479	
				Latin America	Latin America and the Caribbean	4,158	8.9	0.13,00,368	
				Caribbean	Latin America and the Caribbean	4,158	8.9	0.13,00,368	
				Oceania	Oceania	25,605	6.37	0.03,65,506	
				Australia/New Zealand	Australia	25,605	7.68	0.04,47,617	
Fan et al., (34)	The aim of this study was to calculate the average point incidence of PPH in women with placenta previa			Northern America	Northern America	1,692	26.3	0.62,71,017	14
				Asia	Asia	1,545	20.7	0.51,37,526	
				Australia	Australia	1,612	19.2	0.46,55,902	
				Europe	Europe	297	17.8	1.00,34,285	
Moyo, Dzinamarira, Moyo, Murewanhema, & Ross, (7)	To provide an accurate estimation of the prevalence of PPH and to identify regional risk variables.	Google Scholar, EMBASE, Science Direct, CINAHL, MEDLINE, Africa Journals Online (AJOL), SCOPUS, and PubMed	26	Sub-Saharan Africa	Africa	106,640	8.6	0.02,47,569	14
Nigussie, Girma, Molla, Tamir, & Tilahun, (9)	To estimate the pooled magnitude of postpartum haemorrhage and the pooled effect size of the associated factors in Ethiopia	PubMed/MEDLINE online, Science Direct, Hinari, Cochrane Library, CINAHL, African Journals Online, Google and Google Scholars databases	21	Ethiopia	Africa	93,898	8.24	0.025206	15
Tolossa, Fetensa, Zewde, Besho, & Jidha, (8)	To estimates the pooled magnitude of PPH and factors associated with PPH among women who gave birth in Ethiopia	Electronic databases such as Medline, Pub Med, Cochrane library, the Web of Science, and Google Scholar were used to search for articles	9	Ethiopia	Africa	4,032	11.14	0.16,73,791	14
Yoseph Merkeb Alamneh, (40)	to assess the pooled prevalence and associated factors of postpartum haemorrhage among mothers at public health institutions in Ethiopia	MEDLINE/PubMed, EMBASE, Scopus, and Grey literature databases, Google Scholar, Science Direct and Cochrane library were extensively searched.	11	Ethiopia	Africa	16,416	12.5	0.09,35,772	16

**Table 3:** Presents highlights of authors’ conclusions on global postpartum haemorrhage (PPH) measured objectively. It summarizes the total number of included studies, study settings, countries represented, overall sample size, and key conclusions reported by the authors for the period 2000–2025.

Author (year)	Review aim	Search strategy	# No studies	Study setting	Sample size	PPH blood loss >=500 mL	Authors conclusion	Quality AMSTAR-2
Calvert et al., (3)	To provide regional estimates of the prevalence of maternal haemorrhage and explore the effect of methodological differences between studies on any observed regional variation	Google scholar, PubMed, EMBASE, Scopus, Web of Sciences, and Grey literature databases	63	Globally	1,003,694	10.8	Postpartum haemorrhage (PPH) prevalence varies globally, with Africa showing the highest rates and Asia the lowest. Measurement methods and regional differences significantly affect estimates. Visual estimation often underreports blood loss.	12
							Standardizing measurement techniques and improving data collection especially in developing countries is essential for accurate global estimates and better maternal health outcomes. Objective methods are strongly recommended.	
Carroli, Cuesta, Abalos, & Gulmezoglu, (38)	This study conducted a systematic review to assess the global and regional prevalence of postpartum haemorrhage (PPH), aiming to understand its magnitude across diverse settings. Variations remain unclear due to inconsistent data.	Google scholar, PubMed, EMBASE, Scopus, Web of Sciences, and Grey literature databases	120	Globally	3,815,034	6.09	This systematic review analyzed 120 datasets on postpartum haemorrhage (PPH) and 70 on severe PPH (SPPH), covering over 4.3 million women. PPH and SPPH prevalence were approximately 6% and 1.86%, respectively, with significant regional variation.	13
							Reliable global estimates require well-designed studies, standardized measurement methods, and a comprehensive global survey to understand PPH's true magnitude and impact.	
Moyo, Dzinamarira, Moyo, Murewanhema, & Ross, (7)	To provide an accurate estimation of the prevalence of PPH and to identify regional risk variables.	Google Scholar, EMBASE, Science Direct, CINAHL, MEDLINE, Africa Journals Online (AJOL), SCOPUS, and PubMed	26	Sub-Saharan Africa	106,640	8.60	Postpartum haemorrhage (PPH) is the leading cause of maternal death globally, with a significantly higher prevalence in Sub-Saharan Africa. It poses both immediate and long-term health risks. Despite established management strategies, individual risk factors are often unclear.	14
							This systematic review categorizes PPH risk factors into maternal, antenatal, intrapartum, and postpartum groups, highlighting the need for improved identification and prevention efforts.	
Total			209		4,925,368	Average 8.497		

**Table 4:** Presents highlights of authors’ conclusions on global severe postpartum haemorrhage. It summarizes the total number of included studies, study settings, countries represented, overall sample size, and key conclusions reported by the authors for the period 2000–2025.

Author (year)	Review aim	Search strategy	# No studies	Study setting	Sample size	PPH blood loss >=1,000 mL	Authors conclusion	Quality AMSTAR-2
Carroli, Cuesta, Abalos, & Gulmezoglu, (38)	This study conducted a systematic review to assess the global and regional prevalence of postpartum haemorrhage (PPH), aiming to understand its magnitude across diverse settings. Variations remain unclear due to inconsistent data.	Google scholar, PubMed, EMBASE, Scopus, Web of Sciences, and Grey literature databases	70	Globally	505,379	3.04	This systematic review analyzed 70 datasets on 70 on severe PPH (SPPH), covering over 4.3 million women. PPH and SPPH prevalence were approximately 3.04%, objectively measured, with significant regional variation.	13
							Reliable global estimates require well-designed studies, standardized measurement methods, and a comprehensive global survey to understand PPH's true magnitude and impact.	
Huang et al., (35)	To estimate the incidence and identify risk factors of postpartum haemorrhage (PPH) after vaginal delivery	PubMed, Cochrane Library, CINAHL, Web of Science, EMBASE, and ClinicalTrials.gov databases were systematically searched	36	Globally	30,50,532	6%	This review of 36 studies found PPH rates of 6% (≥1,000 mL), identifying 41 risk factors across five categories. Rising global PPH incidence highlights the need for obstetric providers to address labor-related risks prolonged labor, oxytocin use, and genital trauma to reduce maternal morbidity.	13
Total			106		35,55,911	Average 1.55		

### Global pooled prevalence of postpartum haemorrhage (PPH blood loss ≥500 mL) measured non-objectively

This umbrella review synthesized evidence from 12 systematic reviews and meta-analyses, encompassing 319 primary studies conducted across multiple countries. The aggregated data represent a global sample of 8,233,361 women of reproductive age who experienced postpartum haemorrhage (PPH) following childbirth. Based on descriptively the average global prevalence of PPH was 10.625%. These updated findings underscore the substantial global burden of PPH and highlight the urgent need for strengthened maternal health interventions and evidence-based clinical practices (3, 7–9, 34, 35, 38, 40, 41, 43, 44).

**Figure 2:**
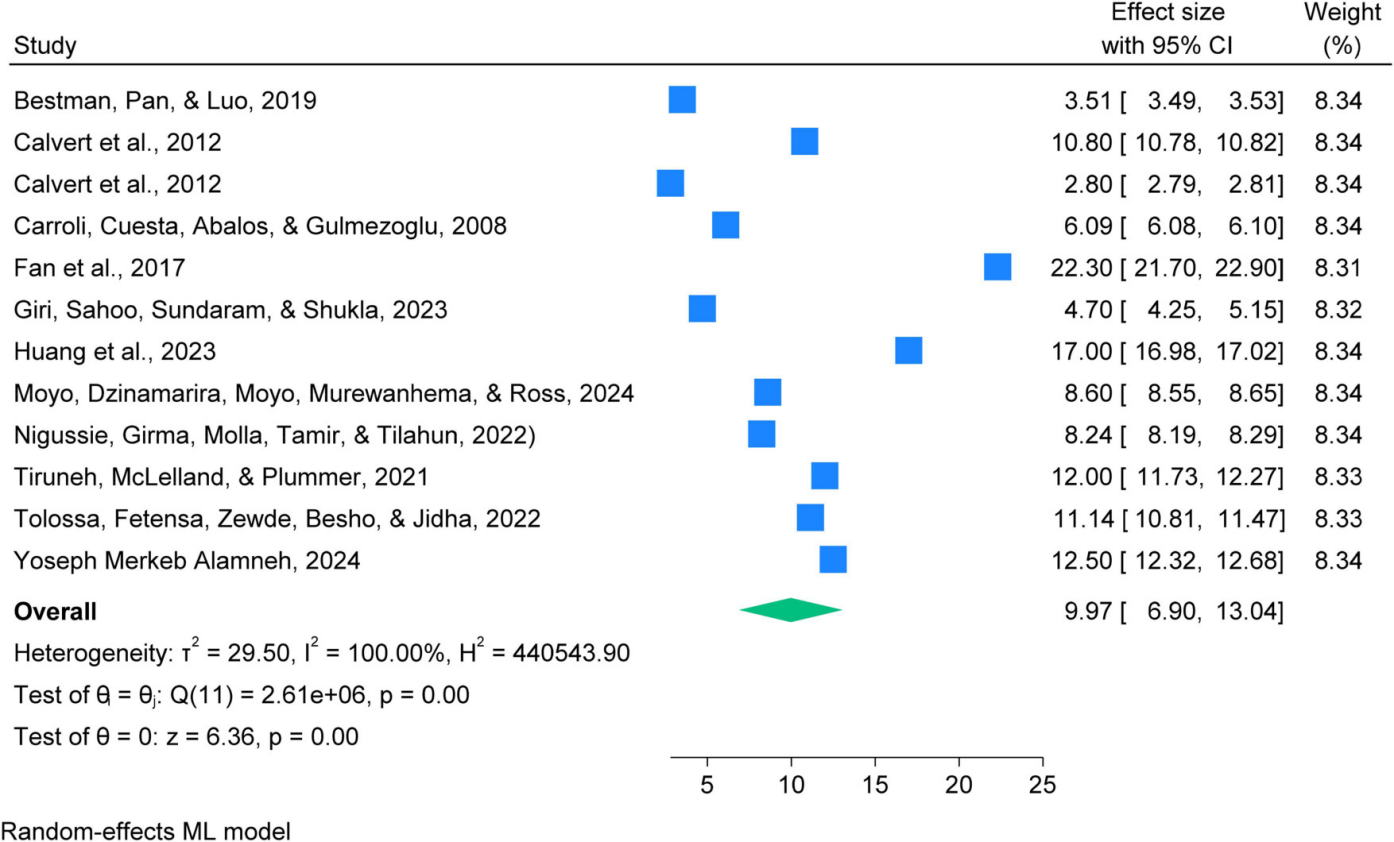
Forest plot showing a global pooled prevalence of postpartum haemorrhage among blood loss ≥500 mL per 100 women giving birth, 2025.

This study found a global pooled prevalence of postpartum haemorrhage was 9.97% (95% CI: 6.90–13.04%), with a statistically significant at (*p* = 0.00) and very high heterogeneity (*I*² = 100.00%). This indicates that approximately 10 out of every 100 women experience PPH globally, highlighting a substantial public health concern (3, 7–9, 34, 35, 38, 40, 41, 43, 44). See details in Figure 2.

### Trends in global prevalence of postpartum haemorrhage (PPH)

This umbrella review analyzed studies grouped by publication year to assess trends in global PPH prevalence (3, 7–9, 34, 35, 38, 40, 41, 43, 44).

**Figure 3:**
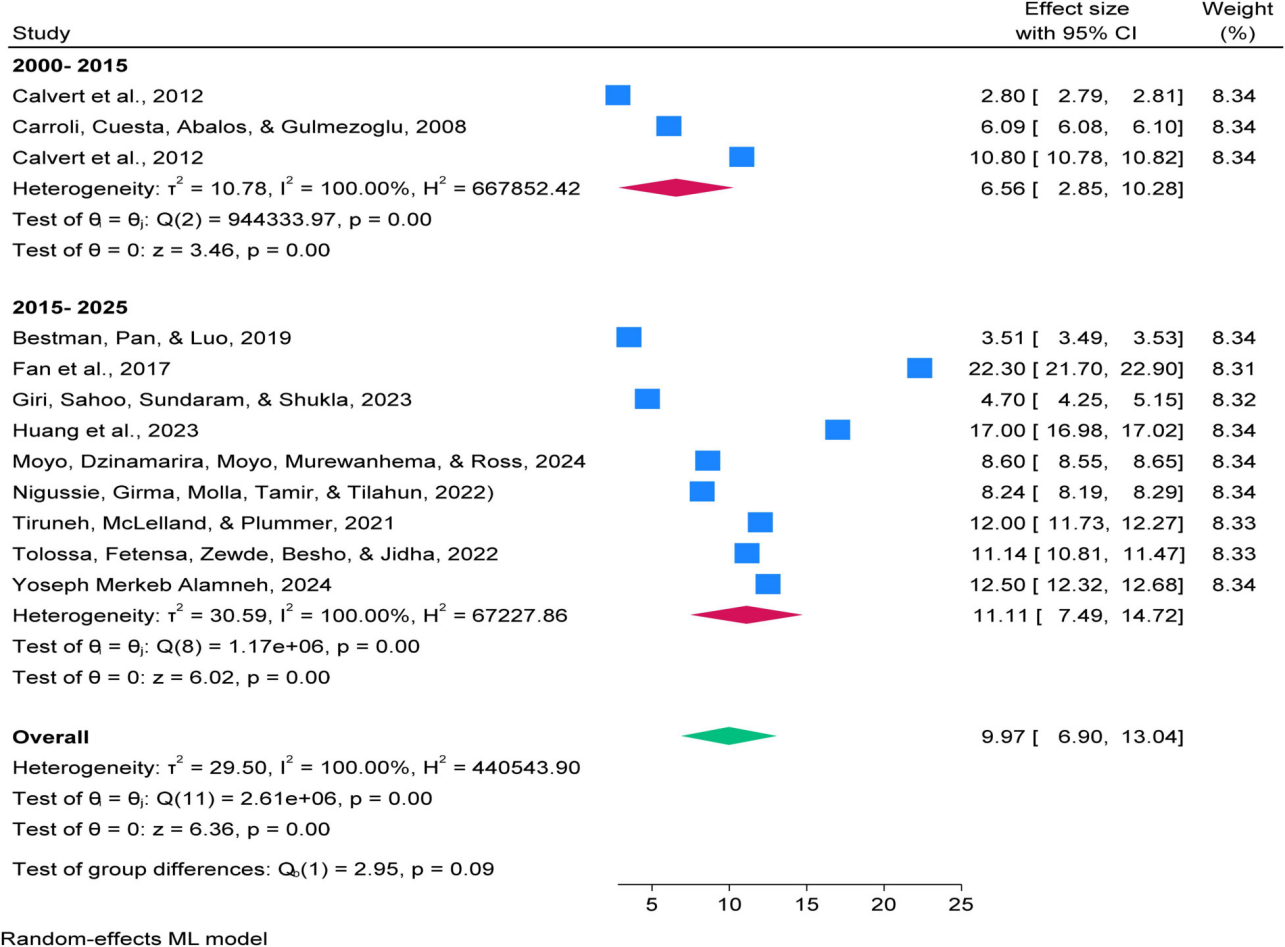
Forest plot showing trends in global prevalence of postpartum haemorrhage (PPH) among blood loss ≥500 mL per 100 women giving birth from 2000 to 2025.

The Global PPH prevalence trends by publication period,between 2000 and 2015, prevalence ranged from 2.80% to 10.80%, with a pooled estimate of 6.56% (95% CI: 2.85–10.28; *I*² = 100%) and (*p* = 0.00). From 2015 to 2025, prevalence varied widely (3.51%−22.30%), with higher estimates in recent studies, indicating increased variability and rising rates. These changes may reflect improved detection, broader geographic coverage, and evolving clinical practices or reporting standards. See details in Figure 3.

### Publication bias assessment

This umbrella review of systematic reviews and meta-analyses estimated the global pooled prevalence of postpartum haemorrhage (PPH), defined as blood loss ≥500 mL. Publication bias was assessed using a funnel plot, which appeared asymmetric and skewed to the left. This visual indication was objectively confirmed by Egger's regression test (*p* = 0.0158), suggesting the presence of publication bias. To adjust for this, a trim-and-fill analysis was conducted, imputing one to three potentially missing studies on the right side of the funnel plot.

**Figure 4:**
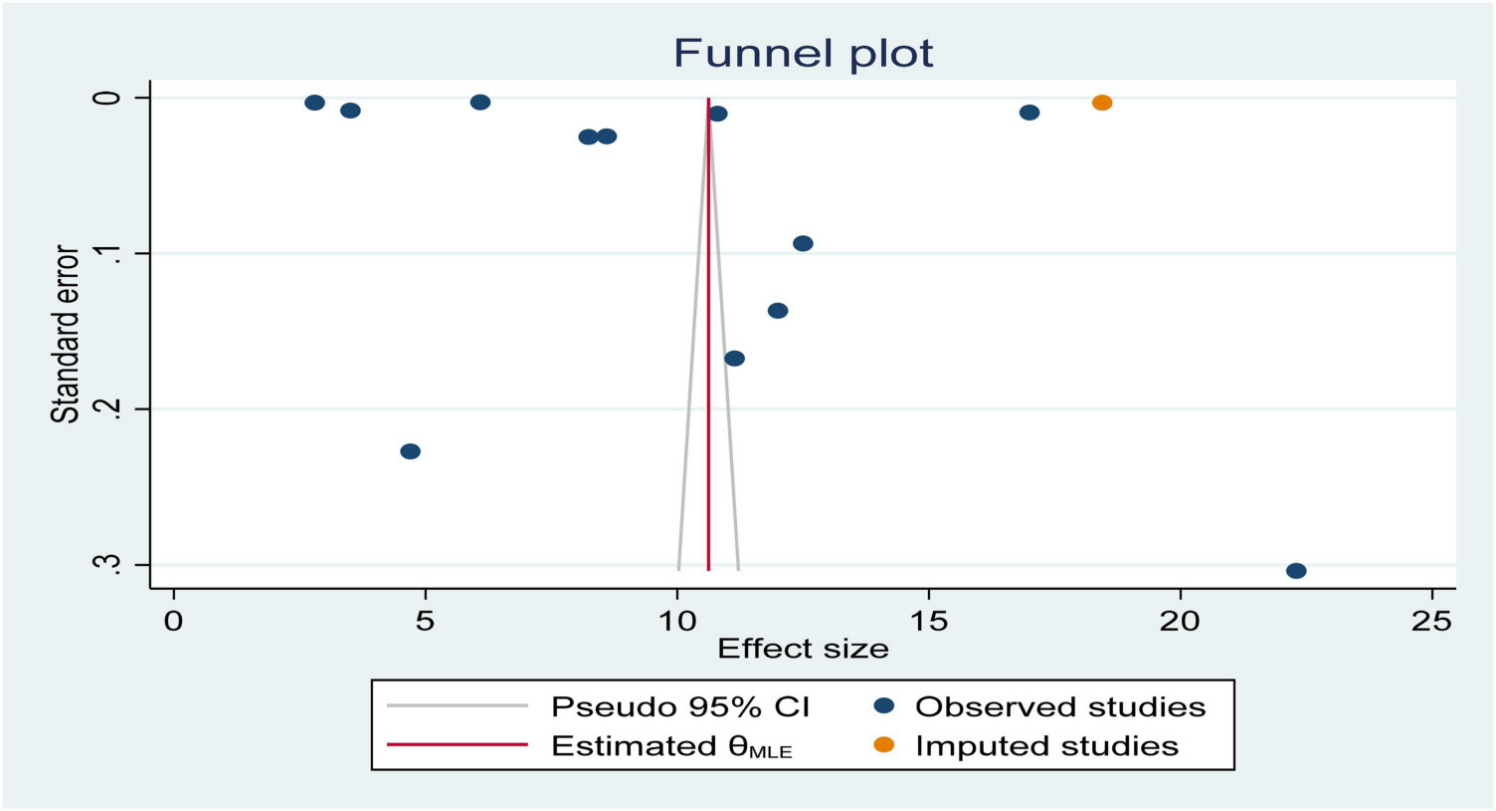
Funnel plot showing symmetric distribution of included studies in global pooled prevalence of postpartum haemorrhage (PPH) among blood loss ≥500 mL per 100 women giving birth from 2000 to 2025.

Following this adjustment, the global pooled prevalence of postpartum haemorrhage (PPH) increased from 9.97% (95% CI: 6.90%–13.05%) to 10.62% (95% CI: 7.53%–13.72%), with both estimates showing narrow confidence intervals, indicating high precision. This suggests that the true effect size may be slightly underestimated when only observed studies are considered. After the trim-and-fill correction, the funnel plot appeared more symmetric, supporting the robustness of the adjusted estimate (see Figure 4).

### Disparities in the burden of postpartum haemorrhage (PPH) across continents

This umbrella review of systematic reviews and meta-analyses demonstrate substantial disparities in the global burden of postpartum haemorrhage (PPH), with prevalence rates varying significantly across continents. These differences are influenced by variations in healthcare infrastructure, maternal care practices, and socioeconomic conditions.

Africa (3, 7–9, 38, 41, 44) and North America (3, 34) report the highest estimated prevalence of PPH. In Africa, this is likely due to limited access to skilled birth attendants, emergency obstetric care, and essential medications. In North America, the wide confidence interval suggests variability across populations and healthcare settings, possibly influenced by disparities in access and maternal risk factors.

Asia (3, 34, 38) and Oceania (3, 38) show the lowest prevalence rates, though Asia's wide confidence interval indicates potential underreporting or data inconsistency.

Australia (34, 38) and Europe (3, 34, 38), despite being high-income regions, show moderate prevalence rates, which may be influenced by clinical definitions, reporting standards, and maternal demographics.

**Figure 5:**
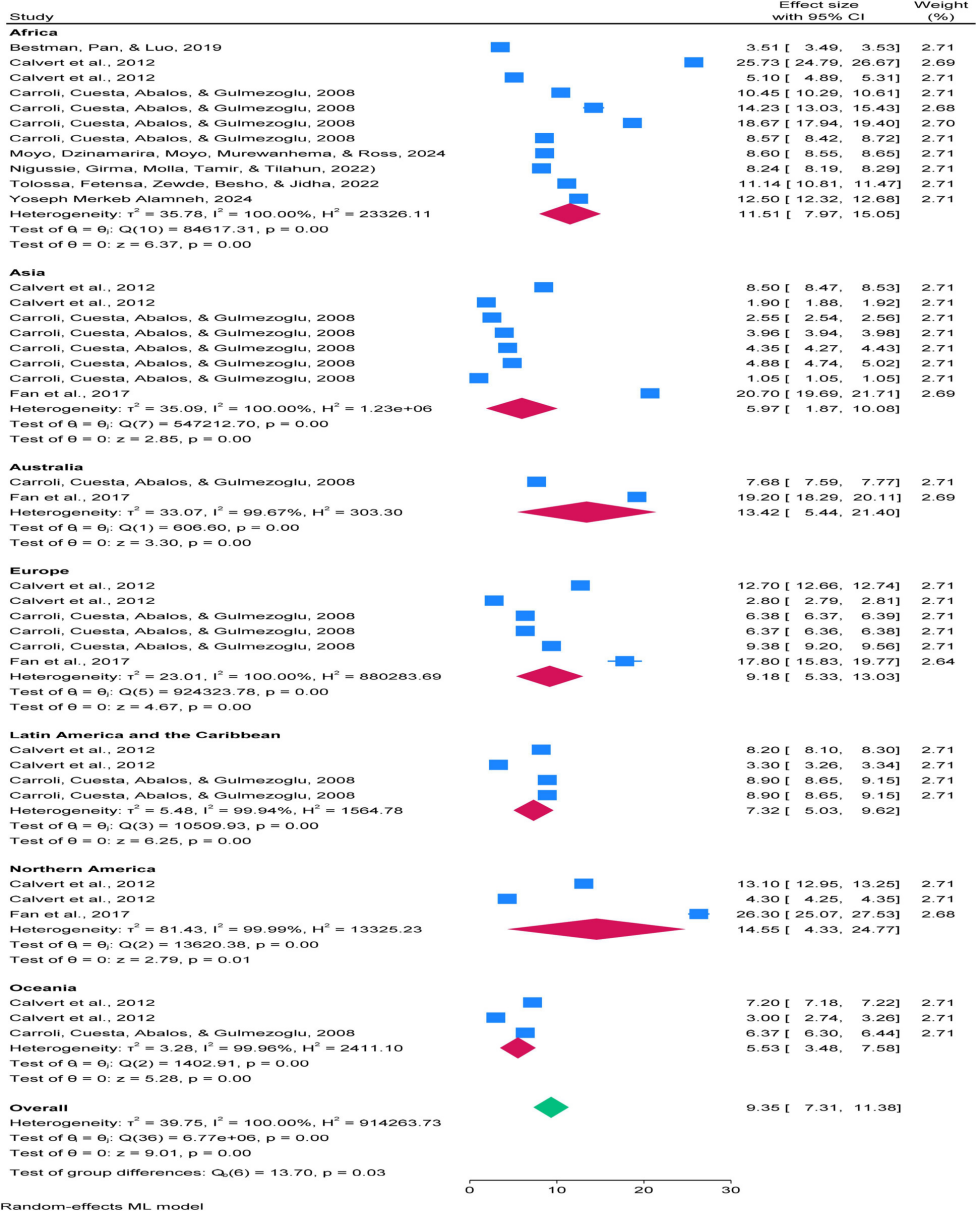
Forest plot showing disparities in the burden of postpartum haemorrhage (PPH) across continents among blood loss ≥500 mL per 100 women giving birth 2025.

Latin America & the Caribbean (3, 38) fall in the mid-range, reflecting progress in maternal health but persistent inequalities in access and quality of care. All included studies underwent rigorous quality assessment using the AMSTAR-2 tool, with scores consistently ranging from 11 to 16, ensuring methodological robustness and reliability of the findings. See detailed information presented in Figure 5 and Supplementary Table S2.

### Subgroup pooled analysis of regional disparities in PPH burden

The subgroup analysis revealed significant regional disparities in the prevalence of postpartum haemorrhage (PPH) across continents. Africa reported a pooled prevalence of 11.51% (95% CI: 7.97–15.05), North America 14.55% (95% CI: 4.33–24.77), and Australia 13.42% (95% CI: 5.44–21.03). Lower estimates were observed in Asia (5.97%), Oceania (5.53%), and Latin America and the Caribbean (7.32%), while Europe reported 9.18%. The overall pooled prevalence was 9.35% (95% CI: 7.31–11.38). These findings underscore marked regional differences, highlighting that PPH remains a global concern rather than a problem confined to low-resource. See details in Figure 5.

### Publication bias assessment

This umbrella review of systematic reviews and meta-analyses estimated the disparities in the burden of postpartum haemorrhage (PPH) across continents, defined as blood loss ≥500 mL. Publication bias was assessed using a funnel plot, which appeared asymmetric and skewed to the left. This visual indication was objectively confirmed by Egger's regression test (*p* = 0.00), suggesting the presence of publication bias. To adjust for this, a trim-and-fill analysis was conducted, imputing six to potentially missing studies on the right side of the funnel plot.

**Figure 6:**
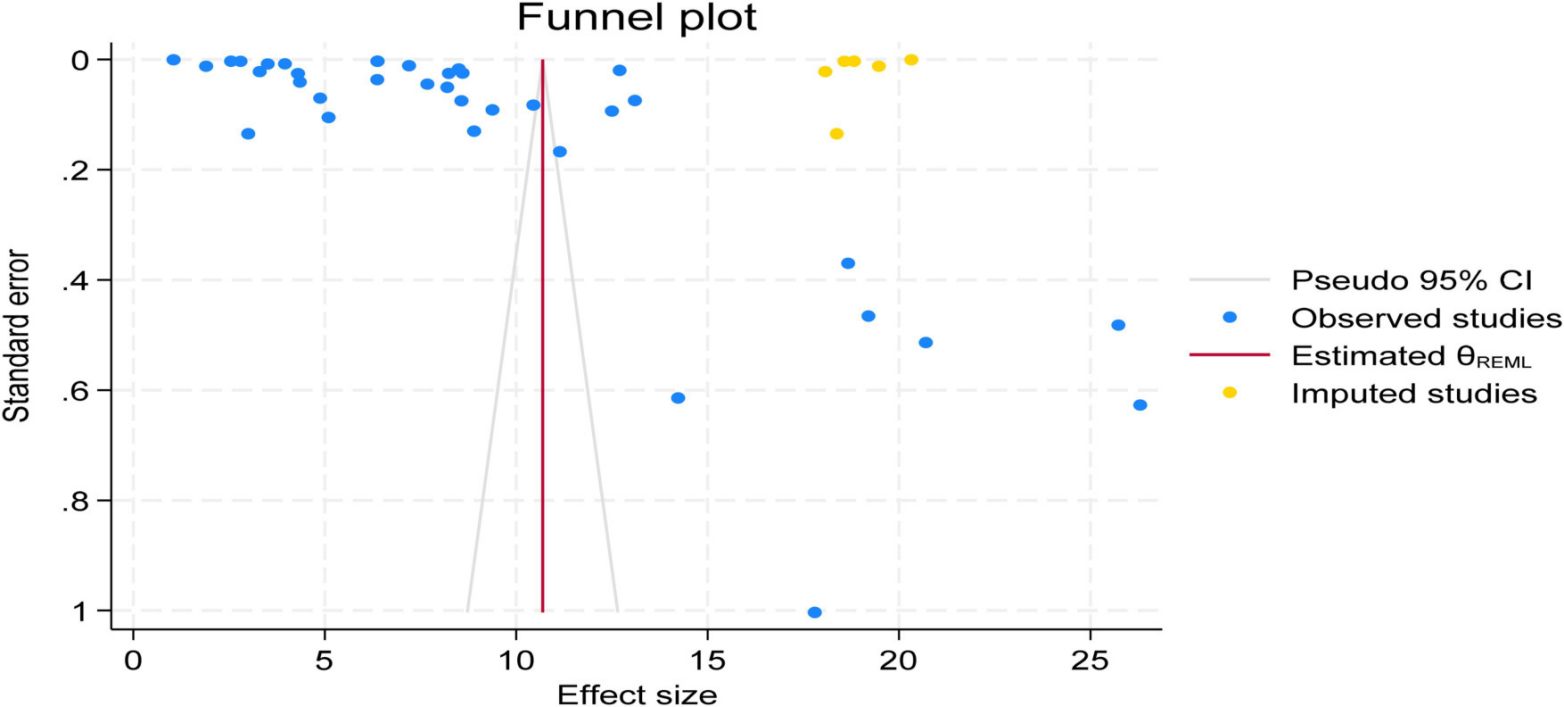
Funnel plot showing symmetric distribution of included studies disparities in the burden of postpartum haemorrhage (PPH) across continents among blood loss ≥500 mL per 100 women giving birth 2025.

Following this adjustment, the global pooled prevalence of postpartum haemorrhage (PPH) increased from 9.347% (95% CI: 7.286−11.409%) to 10.69% (95% CI: 8.65%−12.729%), with both estimates showing narrow confidence intervals, indicating high precision. This suggests that the true effect size may be slightly underestimated when only observed studies are considered. After the trim-and-fill correction, the funnel plot appeared more symmetric, supporting the robustness of the adjusted estimate (see Figure 6).

### Meta-regression findings

Prevalence remains the only significant predictor (coefficient ≈ 1, *p* < 0.001). Year of publication, sample size, and standard error have no effect (*p* = 1.000), indicating no time trend, no small-study bias, and no influence of precision. The model fit is perfect (R² = 100%) with no residual heterogeneity. The prevalence of blood loss ≥500 mL per 100 deliveries is consistent across time, study size, and precision, confirming the robustness and reliability of the findings from this umbrella review.

### Highlights of authors’ conclusions postpartum haemorrhage (PPH) measured objectively

The synthesizes findings from three major systematic reviews and meta-analyses focused on postpartum haemorrhage (PPH), defined as blood loss ≥500 mL. All included studies employed objective blood loss measurement methods, collectively representing data from over 4.9 million women across 209 studies (3, 7, 38).

The descriptive global average prevalence of PPH across all studies is 8.5%, with notable regional variation (3, 7, 38).

Highest Prevalence found in Sub-Saharan Africa reports the highest prevalence at 8.6%, as documented by Moyo et al. (2024), underscoring the region's disproportionate burden (7), and lowest Prevalence: Carroli et al. (2008) found a global average of 6.09%, reflecting variability due to inconsistent data and measurement techniques (38).

The most comprehensive meta-analysis conducted by Calvert et al. (2012) analyzed over 1 million births, reporting a prevalence of 10.8%, and emphasized the critical impact of measurement methods on prevalence estimates (3). All included studies underwent rigorous quality assessment using the AMSTAR-2 tool, with scores consistently ranging from 12 to 14, ensuring methodological robustness and reliability of the findings. See details in Supplementary Table S3.

Global pooled prevalence of postpartum haemorrhage (PPH) measured objectively blood loss ≥500 mL per 100 women giving birth (3, 7, 38), synthesized evidence from three systematic reviews and meta-analyses, encompassing 209 primary studies conducted across multiple countries, and total sample of 4,925,368 women of reproductive age, focusing on the prevalence of postpartum haemorrhage (PPH) (3, 7, 38).

**Figure 7:**
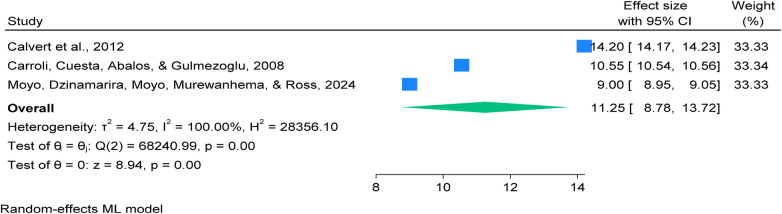
Forest plot showing a global pooled prevalence of postpartum haemorrhage based on objectively measured blood loss ≥500 mL per 100 women giving birth, 2025.

This study found that the global pooled prevalence of PPH 11.25% (95% CI: 8.78–13.72%), with (*p* = 0.00) and heterogeneity (*I*² = 100.00%), based on objectively measured blood loss ≥500 mL per 100 women giving birth. see details in Figure 7.

### Global severe postpartum haemorrhage (SPPH)

Calvert et al. (2012) reported significant global variation in PPH prevalence, with the highest rates observed in Africa and the lowest in Asia. The authors emphasized that visual estimation often underreports blood loss, leading to underestimation of severe PPH (SPPH). They strongly recommended the use of standardized, objective measurement methods to improve the accuracy and comparability of prevalence estimates across regions (3).

Carroli et al. (2008) analyzed 70 primary studies specifically focused on SPPH, covering a population of over 4.3 million women. They reported a global SPPH prevalence of approximately 3.04%. The authors highlighted the urgent need for well-designed studies and a comprehensive global survey to better understand the true burden and impact of SPPH (38). The quality of evidence was assessment using the AMSTAR-2 tool was scored 13 ensuring methodological robustness and reliability of the findings see details in Supplementary Table S4.

### Global pooled prevalence of severe postpartum haemorrhage (SPPH blood loss ≥1,000 mL)

This umbrella review synthesized evidence from two systematic reviews and meta-analyses, encompassing 106 primary studies conducted across multiple countries, and total sample of 3,555,911 women of reproductive age, focusing on the prevalence of postpartum haemorrhage (PPH) (35, 38).

**Figure 8:**
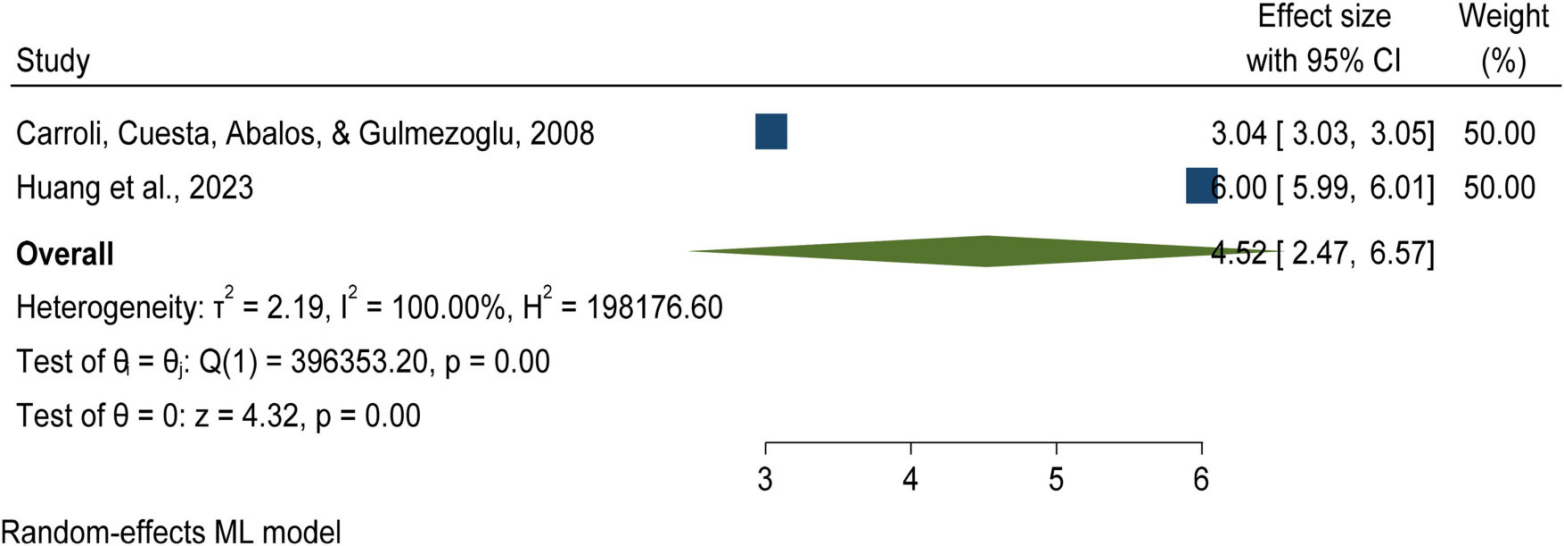
Forest plot showing a global pooled prevalence of severe postpartum haemorrhage among blood loss ≥1,000 mL per 100 women giving birth, 2025.

The global pooled prevalence of severe postpartum haemorrhage (≥1,000 mL blood loss) was 4.52% (95% CI: 2.47%–6.57%), with (*p* = 0.00) and heterogeneity (*I*² = 100.00%), based on studies using non-objective methods to measure blood loss per 100 women giving birth (35, 38) see details in Figure 8.

### Pooled key determinants of postpartum haemorrhage (≥500 mL blood loss)

This umbrella review synthesized evidence on major risk factors for postpartum haemorrhage (PPH). Advanced maternal age significantly increases PPH risk, with women nearly three times more likely to experience PPH compared to younger mothers (AOR = 2.88; 95% CI: 1.80–3.96). Rural residence also poses a high risk (AOR = 3.58; 95% CI: 1.47–5.69), indicating geographic disparities in maternal health outcomes.

Hypertensive disorders during pregnancy elevate PPH risk by 55% (AOR = 1.55; 95% CI: 1.34–1.76). Multiparty is another strong determinant, with women having multiple previous births over three times more likely to develop PPH (AOR = 3.51; 95% CI: 1.71–5.30). Lack of antenatal care (ANC) is the most significant predictor, increasing risk more than fivefold (AOR = 5.29; 95% CI: 2.49–8.09). Similarly, multiple gestation raises risk substantially (AOR = 5.48; 95% CI: 3.23–7.74).

A previous history of PPH strongly predicts recurrence (AOR = 4.70; 95% CI: 2.42–6.98). Caesarean section is associated with a fivefold increase in risk (AOR = 5.40; 95% CI: 2.82–7.98), while prolonged labour nearly triples the likelihood of PPH (AOR = 2.91; 95% CI: 1.32–4.50). Retained placenta is among the most critical factors, increasing risk almost sixfold (AOR = 5.74; 95% CI: 3.55–7.93).

Other notable determinants include perineal trauma (AOR = 1.54; 95% CI: 1.43–1.64), magnesium infusion (AOR = 1.88; 95% CI: 1.61–2.15), and episiotomy (AOR = 2.05; 95% CI: 1.72–2.38). Antepartum haemorrhage (APH) significantly predisposes women to PPH (AOR = 4.38; 95% CI: 1.00–7.77), and stillbirth or intrauterine foetal death (IUFD) increases risk more than threefold (AOR = 3.35; 95% CI: 1.91–4.79). See details in Table 5.

**Table 5:** Pooled key determinants of postpartum haemorrhage among blood loss ≥1,000 mL per 100 women giving birth, 2025.

Authors & Year	Identified determinant	Pooled AOR	95% CI	Heterogeneity	No of studies
(7, 38)	Maternal Residence	3.58	1.47–5.69	*I*² = 100%, *P* = 0.00	2
(7, 9, 35, 38, 40, 44)	Advanced maternal age	2.88	1.80–3.96		6
(9, 35, 38, 40, 42, 44, 46)	Multi-parity	3.51	1.71 –5.30	*I*² = 100%, *P* = 0.00	7
(7, 9, 38, 40, 42, 44, 46)	Absence of anti-natal care visits (ANC)	5.29	2.49–8.09	*I*² = 100%, *P* = 0.00	7
(7, 35, 38, 39, 42)	Multiple gestation	5.48	3.23–7.74	*I*² = 100%, *P* = 0.00	5
(7, 9, 39, 40, 42, 46)	Previous history of PPH	4.70	2.42–6.98	*I*² = 100%, *P* = 0.00	6
(35, 38, 39, 44)	Hypertensive disorder	1.55	1.34–1.76	*I*² = 100%, *P* = 0.00	4
(7, 35, 38)	Nulliparous	4.72	0.83–8.61	*I*² = 100%, *P* = 0.00	3
(38–40, 42)	Caesarean section	5.40	2.82–7.98	*I*² = 99.96%, *P* = 0.00	4
(9, 39)	Prolonged labour	2.91	1.32 –4.50	*I*² = 99.99%, *P* = 0.00	2
(35, 38, 44)	Retained placenta	5.74	3.55–7.93	*I*² = 100%, *P* = 0.00	3
(35, 38, 39, 44)	Perineal Trauma	1.54	1.43–1.64	*I*² = 99.99%, *P* = 0.00	4
(35, 38, 44)	Magnesium infusion	1.88	1.61–2.15	*I*² = 100%, *P* = 0.00	3
(35, 38)	Episiotomy	2.05	1.72–2.38	*I*² = 99.99%, *P* = 0.00	2
(7, 42)	Antepartum Haemorrhage (APH)	4.38	1.00–7.77	*I*² = 100%, *P* = 0.00	2
(7, 42)	Intrauterine foetal death (IUFD)	3.35	1.91–4.79	*I*² = 99.95%, *P* = 0.00	2

## Discussion

Postpartum haemorrhage (PPH) remains the leading cause of maternal mortality worldwide, despite being both preventable and treatable. This umbrella review of systematic reviews and meta-analyses highlights significant global disparities in the burden of postpartum haemorrhage (PPH), identifying key pooled determinants. The evidence shows that nearly 1 in 10 women experience PPH, with prevalence rising to over 11% when objective blood loss measurements are applied. These findings are strongly supported by the World Health Organization, which reports that PPH accounts for 27% of maternal deaths globally and occurs in 5%–10% of deliveries. Uterine atony is responsible for approximately 70% of cases (4). These findings call for urgent, targeted interventions and the adoption of standardized clinical protocols to mitigate risks and improve maternal outcomes across diverse healthcare systems.

The current umbrella review reveals that severe postpartum haemorrhage (PPH) affects approximately 1 in 20 women giving birth, exposing a critical gap in maternal care globally. This finding aligns with existing WHO policy and strategic direction. Despite being preventable and treatable, PPH remains the leading cause of maternal death, accounting for 27% of global maternal mortality (4). In response, WHO and its partners launched a strategic Roadmap (2023–2030) outlining goals in research, standards, implementation, and advocacy (5). The framework prioritizes high-burden countries and calls for urgent investment in health system strengthening and evidence-based interventions to accelerate progress toward SDG target 3.1 and ensure safer childbirth for all women (5).

This umbrella review identified key risk factors for postpartum haemorrhage (PPH), including advanced maternal age, lack of antenatal care, obstetric complications, multiparty, and rural residence. These findings are strongly supported by previous systematic reviews and meta-analyses, which consistently associate PPH with factors such as prolonged labour, twin pregnancy, antepartum haemorrhage, and induction of labour (7–9). Together, these insights underscore the urgent need for targeted prevention strategies, improved antenatal care attendance, and close monitoring of high-risk pregnancies to reduce PPH-related morbidity and enhance maternal health outcomes, especially in resource-limited settings. And also according to FIGO, all birth attendants especially in low- and middle-income countries must be equipped with appropriate medications and trained in PPH prevention and management (48). Routine active management of the third stage of labour and physiologic care are essential to reduce PPH incidence and avoid costly, life-saving surgical interventions (48). This umbrella review synthesizes global evidence to resolve inconsistencies, guide policy, and advance maternal health equity and clinical decision-making worldwide. The findings strongly reinforce the WHO consolidated guidelines, which provide 51 actionable recommendations for PPH prevention, diagnosis, treatment, and health system interventions, including 20 new or updated directives. Organized across antenatal, intrapartum, postpartum, and emergency care contexts, these evidence-based strategies empower policymakers and clinicians to standardize care, strengthen health systems, and reduce maternal mortality. Clear classification and implementation guidance ensure practical application, driving progress toward SDG 3.1 and WHO's 2023–2030 maternal health roadmap (49).

## Strengths and Limitations of the Umbrella Review

### Strength

This umbrella review provides a robust and comprehensive synthesis of global systematic reviews on postpartum haemorrhage (PPH), consolidating fragmented evidence to identify key risk factors such as maternal age, lack of antenatal care, obstetric complications, and rural residence. By integrating high-quality evidence (AMSTAR-2 scores ranging from 11 to 16), the review ensures methodological rigor and reliability. It aligns with WHO and FIGO recommendations, offering policy-relevant insights for improving maternal care worldwide. Importantly, the prevalence of blood loss ≥500 mL per 100 deliveries remains consistent across time, study size, and precision, confirming the robustness and generalizability of the findings. This synthesis not only strengthens the evidence base but also highlights research gaps and informs actionable strategies to reduce maternal morbidity and mortality globally.

### Limitations

While this umbrella review provides a comprehensive global synthesis of systematic reviews on postpartum haemorrhage (PPH), several limitations should be acknowledged. First, the findings depend on the methodological rigor of the included reviews, which may vary despite quality appraisal using AMSTAR-2. Second, variability in PPH definitions and measurement techniques across studies may affect comparability and interpretation of pooled estimates. Third, the exclusion of non-English publications introduces language bias, potentially limiting the global representativeness of the evidence. Fourth, the use of aggregate data may lead to ecological fallacy, as associations observed at the review level may not reflect individual-level relationships. Finally, publication bias cannot be fully ruled out, as studies with significant results are more likely to be published. These limitations highlight the need for standardized definitions, inclusive language policies, and more granular data in future research to strengthen evidence-based maternal health interventions.

### Implications for public health and policy

The high global prevalence of postpartum haemorrhage (PPH) demands urgent policy action.

Preventing postpartum haemorrhage (PPH) is both urgent and achievable. Timely, evidence-based interventions can save thousands of lives every year. As WHO emphasizes, “PPH is preventable every maternal death represents a systemic failure”. Aligning with WHO's 2023–2030 Roadmap and SDG 3.1, decisive policy action is essential to reduce maternal mortality and advance health equity globally.

Strengthening care before, during, and after childbirth must be a priority.

Governments and health systems should integrate routine PPH risk screening into antenatal care, ensure access to skilled birth attendants, and strengthen emergency obstetric services, especially in rural and low-resource settings.

Policies must strengthen continuous training for healthcare providers, ensure resilient supply chains for uterotonics and essential medicines, and guarantee blood transfusion readiness with robust emergency response systems to effectively prevent and manage postpartum haemorrhage and reduce maternal mortality globally.

Further research is needed to refine context-specific interventions, improve objective measurement of blood loss, and evaluate preventive strategies.

Longitudinal studies and implementation research can guide scalable solutions to reduce PPH-related morbidity and mortality globally.

Every year, thousands of mothers die from PPH a tragedy that is both preventable and unacceptable. These are not just statistics; they represent families and futures lost. Immediate policy action is essential to guarantee timely, effective care and to uphold maternal health as a fundamental human right. As the global call reminds us: “No woman should die giving life”.

## Conclusion and recommendation

This umbrella review highlights postpartum haemorrhage (PPH) as a major global health concern, affecting approximately 10% of women, with higher rates (11%) observed in settings using objective blood loss measurements. Notably, severe PPH affects nearly 1 in 20 women, indicating a substantial risk of life-threatening bleeding after childbirth. These findings underscore the urgent need for targeted interventions to improve maternal outcomes globally. Despite being preventable, low- and middle-income countries (LMICs) continue to bear the highest burden an urgent call for targeted interventions, strengthened health systems, and equitable resource allocation to eliminate this avoidable cause of maternal mortality. Postpartum haemorrhage (PPH) is influenced by multiple demographic, obstetric, and clinical factors. High-risk groups include women of advanced age, rural residence, multiparty, hypertensive disorders, and those without antenatal care. Obstetric complications such as multiple gestation, previous PPH, Caesarean section, prolonged labour, retained placenta, antepartum haemorrhage, and stillbirth further elevate risk. These findings emphasize the need for targeted interventions, improved ANC coverage, and vigilant intrapartum monitoring to reduce maternal morbidity and mortality. Policymakers should prioritize universal access to antenatal care, skilled birth attendance, and timely emergency obstetric services. Integrating risk-based screening and PPH prevention into national maternal health strategies, especially in rural and low-resource settings, is essential. Strengthening health systems, training frontline providers, and ensuring availability of uterotonics and blood transfusion services will reduce PPH-related morbidity and mortality and promote maternal health equity.

## Data Availability

The original contributions presented in the study are included in the article/Supplementary Material, further inquiries can be directed to the corresponding author.

## References

[1] Organization WH. World Health Organization. Sexual and Reproductive Health and Research (SRH). Human Reproduction Program (HRP) Research for Impact (2006). Available online at: https://www.who.int/teams/sexual-and-reproductive-health-and-research/key-areas-of-work/sexual-health/defining-sexual-health (Accessed March 30, 2023).

[2] Goffinet F Mercier F Teyssier V Pierre F Dreyfus M Mignon A Postpartum haemorrhage: recommendations for clinical practice by the CNGOF 2004. Gynecol Obstet Fertil. (2005) 33(4):268–74. 10.1016/j.gyobfe.2005.03.016 15894217

[3] Calvert C Thomas SL Ronsmans C Wagner KS Adler AJ Filippi V. Identifying regional variation in the prevalence of postpartum haemorrhage: a systematic review and meta-analysis. PLoS One. (2012) 7(7):e41114. 10.1371/journal.pone.0041114 22844432 PMC3402540

[4] Organization WH. Trends in Maternal Mortality 2000 to 2020: Estimates by WHO, UNICEF, UNFPA, World Bank Group and UNDESA/Population Division (2023). World Health Organization.

[5] Organization WH. A Roadmap to Combat Postpartum Haemorrhage Between 2023 and 2030. Geneva: World Health Organization (WHO) (2023).

[6] Fang P Sun T Liang Z Liu L Zhang Z Yang Y. Global, regional, and national trends in maternal hemorrhage, 1992–2021: a hierarchical cluster and age-period-cohort analysis of the global burden of disease study 2021 and projections to 2036. BMC Public Health. (2025) 25(1):2540. 10.1186/s12889-025-23738-5 40707872 PMC12288340

[7] Moyo E Dzinamarira T Moyo P Murewanhema G Ross A. Magnitude and determinants of postpartum hemorrhage in Sub-Saharan Africa: a systematic review and meta-analysis. Clin Exp Obstet Gynecol. (2024) 51(10):229. 10.31083/j.ceog5110229

[8] Tolossa T Fetensa G Zewde EA Besho M Jidha TD. Magnitude of postpartum hemorrhage and associated factors among women who gave birth in Ethiopia: a systematic review and meta-analysis. Reprod Health. (2022) 19(1):194. 10.1186/s12978-022-01498-4 36131345 PMC9490897

[9] Nigussie J Girma B Molla A Tamir T Tilahun R. Magnitude of postpartum hemorrhage and its associated factors in Ethiopia: a systematic review and meta-analysis. Reprod Health. (2022) 19(1):63. 10.1186/s12978-022-01360-7 35264188 PMC8905908

[10] Valentine JC Pigott TD Rothstein HR. How many studies do you need? A primer on statistical power for meta-analysis. J Educ Behav Stat. (2010) 35(2):215–47. 10.3102/1076998609346961

[11] Aromataris E Fernandez R Godfrey CM Holly C Khalil H Tungpunkom P. Summarizing systematic reviews: methodological development, conduct and reporting of an umbrella review approach. JBI Evid Implement. (2015) 13(3):132–40. 10.1097/XEB.0000000000000055 26360830

[12] Shea BJ Reeves BC Wells G Thuku M Hamel C Moran J AMSTAR 2: a critical appraisal tool for systematic reviews that include randomised or non-randomised studies of healthcare interventions, or both. Br Med J. (2017) 358:j4008. 10.1136/bmj.j4008 28935701 PMC5833365

[13] Higgins JP Thompson SG. Quantifying heterogeneity in a meta-analysis. Stat Med. (2002) 21(11):1539–58. 10.1002/sim.1186 12111919

[14] DerSimonian R Laird N. Meta-analysis in clinical trials. Control Clin Trials. (1986) 7(3):177–88. 10.1016/0197-2456(86)90046-2 3802833

[15] Chandler J Cumpston M Li T Page MJ Welch V. Cochrane Handbook for Systematic Reviews of Interventions. Hoboken: Wiley (2019). p. 4. 10.1002/14651858.ED000142 PMC10284251 31643080

[16] Omotayo MO Abioye AI Kuyebi M Eke AC. Prenatal anemia and postpartum hemorrhage risk: a systematic review and meta-analysis. J Obstet Gynaecol Res. (2021) 47(8):2565–76. 10.1111/jog.14834 34002432 PMC9258034

[17] Suarez S Conde-Agudelo A Borovac-Pinheiro A Suarez-Rebling D Eckardt M Theron G Uterine balloon tamponade for the treatment of postpartum hemorrhage: a systematic review and meta-analysis. Am J Obstet Gynecol. (2020) 222(4):293.e1–52. 10.1016/j.ajog.2019.11.1287 31917139

[18] Maswime S Buchmann E. A systematic review of maternal near miss and mortality due to postpartum hemorrhage. Int J Gynaecol Obstet. (2017) 137(1):1–7. 10.1002/ijgo.12096 28099749

[19] Desta M Amha H Anteneh Bishaw K Adane F Assemie MA Kibret GD Prevalence and predictors of uterine rupture among Ethiopian women: a systematic review and meta-analysis. PLoS One. (2020) 15(11):e0240675. 10.1371/journal.pone.0240675 33137135 PMC7605683

[20] Azeze GA Kassie GA Lombebo AA Efa AG Asgedom YS Negash BT Non-pneumatic anti-shock garment utilization and associated factors in Ethiopia: a systematic review and meta-analysis. BMC Pregnancy Childbirth. (2025) 25(1):187. 10.1186/s12884-025-07299-4 39979826 PMC11844103

[21] Pal K Sadanandan DM Gupta A Nayak D Pyakurel M Keepanasseril A Maternal and perinatal outcome in pregnancies complicated with portal hypertension: a systematic review and meta-analysis. Hepatol Int. (2023) 17(1):170–9. 10.1007/s12072-022-10385-w 35802227

[22] Ruiz MT Azevedo NF de Resende CV Rodrigues WF Meneguci J Contim D Quantification of blood loss for the diagnosis of postpartum hemorrhage: a systematic review and meta-analysis. Rev Bras Enferm. (2023) 76(6):e20230070. 10.1590/0034-7167-2023-0070 38055493 PMC10695064

[23] Neary C Naheed S McLernon DJ Black M. Predicting risk of postpartum haemorrhage: a systematic review. Bjog. (2021) 128(1):46–53. 10.1111/1471-0528.16379 32575159

[24] Abneh AA Kassie TD Gelaw SS. The magnitude and associated factors of immediate postpartum anemia among women who gave birth in Ethiopia: systematic review and meta-analysis, 2023. BMC Pregnancy Childbirth. (2024) 24(1):317. 10.1186/s12884-024-06495-y 38664625 PMC11044590

[25] Yao X Shan SS Li YH Ding LJ Wan Y Zhao YY Roles and challenges encountered by midwives in the management of postpartum haemorrhage following normal vaginal delivery: a scoping review. Nurs Open. (2024) 11(6):e2221. 10.1002/nop2.2221 38923309 PMC11194447

[26] Makino Y Miyake K Okada A Ikeda Y Okada Y. Predictive accuracy of the shock index for severe postpartum hemorrhage in high-income countries: a systematic review and meta-analysis. J Obstet Gynaecol Res. (2022) 48(8):2027–37. 10.1111/jog.15292 35661488

[27] Parry Smith WR Papadopoulou A Thomas E Tobias A Price MJ Meher S Uterotonic agents for first-line treatment of postpartum haemorrhage: a network meta-analysis. Cochrane Database Syst Rev. (2020) 11(11):Cd012754. 10.1002/14651858.CD012754.pub2 33232518 PMC8130992

[28] Wang T Li H Liu Y Min XK. Quantitative blood loss measurement methods for early detection of primary postpartum haemorrhage following vaginal birth: a scoping review. J Clin Nurs. (2024) 33(10):3869–85. 10.1111/jocn.17216 38764248

[29] Okunlola O Raza S Osasan S Sethia S Batool T Bambhroliya Z Race/ethnicity as a risk factor in the development of postpartum hemorrhage: a thorough systematic review of disparity in the relationship between pregnancy and the rate of postpartum hemorrhage. Cureus J Med Sci. (2022) 14(6):e26460. 10.7759/cureus.26460 PMC9339374 35923676

[30] de Moreuil C Mehic D Nopp S Kraemmer D Gebhart J Schramm T Hemostatic biomarkers associated with postpartum hemorrhage: a systematic review and meta-analysis. Blood Adv. (2023) 7(19):5954–67. 10.1182/bloodadvances.2023010143 37307172 PMC10562765

[31] Franchini M Mengoli C Cruciani M Bergamini V Presti F Marano G Safety and efficacy of tranexamic acid for prevention of obstetric haemorrhage: an updated systematic review and meta-analysis. Blood Transfus. (2018) 16(4):329–37. 10.2450/2018.0026-18 29757132 PMC6034773

[32] Pileggi-Castro C Nogueira-Pileggi V Tunçalp Ö Oladapo OT Vogel JP Souza JP. Non-pneumatic anti-shock garment for improving maternal survival following severe postpartum haemorrhage: a systematic review. Reprod Health. (2015) 12:28. 10.1186/s12978-015-0012-0 25889868 PMC4422609

[33] Addisu D Gebeyehu NA Belachew YY Mekie M. Utilization of non-pneumatic anti-shock garment for treating obstetric hemorrhage and associated factors among obstetric care providers in Ethiopia: a systematic review and meta-analysis. PLoS One. (2023) 18(11):e0294052. 10.1371/journal.pone.0294052 37972081 PMC10653477

[34] Fan DZ Xia Q Liu L Wu SZ Tian G Wang W The incidence of postpartum hemorrhage in pregnant women with placenta previa: a systematic review and meta-analysis. PLoS One. (2017) 12(1):e0170194. 10.1371/journal.pone.0170194 28107460 PMC5249070

[35] Huang CR Xue B Gao Y Yue SW Redding SR Wang R Incidence and risk factors for postpartum hemorrhage after vaginal delivery: a systematic review and meta-analysis. J Obstet Gynaecol Res. (2023) 49(7):1663–76. 10.1111/jog.15654 37069822

[36] Schoretsanitis G Gastaldon C Ochsenbein-Koelble N Olbrich S Barbui C Seifritz E. Postpartum hemorrhage and postpartum depression: a systematic review and meta-analysis of observational studies. Acta Psychiatr Scand. (2024) 150(5):274–83. 10.1111/acps.13583 37286177

[37] Feduniw S Warzecha D Szymusik I Wielgos M. Epidemiology, prevention and management of early postpartum hemorrhage—a systematic review. Ginekol Pol. (2020) 91(1):38–44. 10.5603/GP.2020.0009 32039467

[38] Carroli G Cuesta C Abalos E Gulmezoglu AM. Epidemiology of postpartum haemorrhage: a systematic review. Best Pract Res Clin Obstet Gynaecol. (2008) 22(6):999–1012. 10.1016/j.bpobgyn.2008.08.004 18819848

[39] Ende HB Lozada MJ Chestnut DH Osmundson SS Walden RL Shotwell MS Risk factors for atonic postpartum hemorrhage: a systematic review and meta-analysis. Obstet Gynecol. (2021) 137(2):305–23. 10.1097/AOG.0000000000004228 33417319 PMC8336570

[40] Yoseph Merkeb Alamneh FA. Postpartum hemorrhage and associated factors among mothers who delivered at public health institutions in Ethiopia: a systematic review and meta-analysis. Curr Pediatr Res. (2024) 28(03):2184–8. 10.35841/0971-9032.28.03.2184-2188

[41] Tiruneh BT McLelland G Plummer V. 325 the incidence of primary postpartum haemorrhage following in-hospital birth: a systematic review and meta-analysis. Int J Epidemiol. (2021) 50(Supplement_1). 10.1093/ije/dyab168.661

[42] Yunas I Islam MA Sindhu KN Devall AJ Podesek M Alam SS Causes of and risk factors for postpartum haemorrhage: a systematic review and meta-analysis. Lancet. (2025) 405(10488):1468–80. 10.1016/S0140-6736(25)00448-9 40188841

[43] Giri S Sahoo S Sundaram S Shukla A. Maternal and fetal outcomes in pregnant patients with non-cirrhotic portal hypertension: a systematic review and meta-analysis. Obstet Med. (2023) 16(3):170–7. 10.1177/1753495X221143864 37719996 PMC10504878

[44] Bestman PL Pan X Luo J. The prevalernce and risk factors of post-partum haemorrhage in Africa: a systematic review. Res Sq. (2019). 10.21203/rs.2.19608/v1

[45] Durmaz A Komurcu N. Relationship between maternal characteristics and postpartum hemorrhage: a meta-analysis study. J Nurs Res. (2018) 26(5):362–72. 10.1097/jnr.0000000000000245 29219937

[46] Nurfauzia YP. Risk factors for atonic postpartum hemorrhage: a systematic review. JAdv Res Med Health Sci. (2023) 9(8):47–54. 10.53555/nnmhs.v9i8.1790

[47] Page MJ McKenzie JE Bossuyt PM Boutron I Hoffmann TC Mulrow CD The PRISMA 2020 statement: an updated guideline for reporting systematic reviews. Br Med J. (2021) 372:n71. 10.1136/bmj.n71 33782057 PMC8005924

[48] Escobar MF Nassar AH Theron G Barnea ER Nicholson W Ramasauskaite D FIGO recommendations on the management of postpartum hemorrhage 2022. Int J Gynaecol Obstet. (2022) 157(Suppl 1):3–50. 10.1002/ijgo.14116 35297039 PMC9313855

[49] Organization WH. Consolidated Guidelines for the Prevention, Diagnosis and Treatment of Postpartum Haemorrhage. Consolidated Guidelines for the Prevention, Diagnosis and Treatment of Postpartum Haemorrhage (2025).

