# Supplementary material for: Global burden, disparities, and determinants of postpartum haemorrhage among women who gave birth: an umbrella review of systematic reviews and meta-analyses
Source: Front Reprod Health. 2025 Dec 18;7:1721550. doi: 10.3389/frph.2025.1721550 (PMC12756386; doi:10.3389/frph.2025.1721550)
Supplement: Supplementary file 3 [file Table2.docx]

S1: Search strategy and lists of included and excluded studies for postpartum haemorrhage among women who gave birth History and Search Details

| **Search** | **Actions** | **Details** | **Query** | **Results** | **Time** |
| --- | --- | --- | --- | --- | --- |
| #41 |  |  | Search: **("Prevalence"[Mesh] AND ((((("Postpartum Hemorrhage"[Mesh]) OR (( "Postpartum Hemorrhage/epidemiology"[Majr] OR "Postpartum Hemorrhage/etiology"[Majr] )))** Filters: **Full text, Meta-Analysis, Review, Systematic Review, English, Humans** | 10 | 04:10:52 |
| #42 |  |  | Search: **("Prevalence"[Mesh] AND ((((("Postpartum Hemorrhage"[Mesh]) OR (( "Postpartum Hemorrhage/epidemiology"[Majr] OR "Postpartum Hemorrhage/etiology"[Majr] )))** Filters: **Full text, Meta-Analysis, Systematic Review, English, Humans** | 6 | 04:10:27 |
| #40 |  |  | Search: **("Prevalence"[Mesh] AND ((((("Postpartum Hemorrhage"[Mesh]) OR (( "Postpartum Hemorrhage/epidemiology"[Majr] OR "Postpartum Hemorrhage/etiology"[Majr] )))** Filters: **Full text, Meta-Analysis, Review, Systematic Review, English, Humans, Female** | 9 | 04:09:32 |
| #39 |  |  | Search: **("Prevalence"[Mesh] AND ((((("Postpartum Hemorrhage"[Mesh]) OR (( "Postpartum Hemorrhage/epidemiology"[Majr] OR "Postpartum Hemorrhage/etiology"[Majr] )))** Filters: **Full text, Associated data, Meta-Analysis, Review, Systematic Review, English, Humans, Female** | 3 | 04:09:19 |
| #38 |  |  | Search: **("Prevalence"[Mesh] AND ((((("Postpartum Hemorrhage"[Mesh]) OR (( "Postpartum Hemorrhage/epidemiology"[Majr] OR "Postpartum Hemorrhage/etiology"[Majr] ))) OR ("Pregnancy Complications"[Majr])) AND ((meta-analysis[Filter] OR review[Filter] OR systematicreview[Filter]) AND (fft[Filter]) AND (humans[Filter]) AND (female[Filter]) AND (data[Filter]) AND (english[Filter])))** Filters: **Full text, Associated data, Meta-Analysis, Review, Systematic Review, English, Humans, Female** | 261 | 04:08:06 |
| #37 |  |  | Search: **("Prevalence"[Mesh] OR AND ((((("Postpartum Hemorrhage"[Mesh]) OR (( "Postpartum Hemorrhage/epidemiology"[Majr] OR "Postpartum Hemorrhage/etiology"[Majr] ))) OR ("Pregnancy Complications"[Majr])) AND ((meta-analysis[Filter] OR review[Filter] OR systematicreview[Filter]) AND (fft[Filter]) AND (humans[Filter]) AND (female[Filter]) AND (data[Filter]) AND (english[Filter])))** Filters: **Full text, Associated data, Meta-Analysis, Review, Systematic Review, English, Humans, Female** | 10,772 | 04:07:43 |
| #36 |  |  | Search: **("Prevalence"[Mesh] OR "Epidemiology"[Mesh] OR "epidemiology" [Subheading]) AND ((((("Postpartum Hemorrhage"[Mesh]) OR (( "Postpartum Hemorrhage/epidemiology"[Majr] OR "Postpartum Hemorrhage/etiology"[Majr] ))) OR ("Pregnancy Complications"[Majr])) AND ((meta-analysis[Filter] OR review[Filter] OR systematicreview[Filter]) AND (fft[Filter]) AND (humans[Filter]) AND (female[Filter]) AND (data[Filter]) AND (english[Filter])))** Filters: **Full text, Associated data, Meta-Analysis, Review, Systematic Review, English, Humans, Female** | 2,230 | 04:07:18 |
| #35 |  |  | Search: **("Prevalence"[Mesh] OR "Epidemiology"[Mesh] OR "epidemiology" [Subheading]) AND ((((("Postpartum Hemorrhage"[Mesh]) OR (( "Postpartum Hemorrhage/epidemiology"[Majr] OR "Postpartum Hemorrhage/etiology"[Majr] ))) OR ("Pregnancy Complications"[Majr])) AND ((meta-analysis[Filter] OR review[Filter] OR systematicreview[Filter]) AND (fft[Filter]) AND (humans[Filter]) AND (female[Filter]) AND (data[Filter]) AND (english[Filter])))** Filters: **Full text, Associated data, Meta-Analysis, Review, Systematic Review, English, Humans** | 2,230 | 04:07:06 |
| #34 |  |  | Search: **("Prevalence"[Mesh] OR "Epidemiology"[Mesh] OR "epidemiology" [Subheading]) AND ((((("Postpartum Hemorrhage"[Mesh]) OR (( "Postpartum Hemorrhage/epidemiology"[Majr] OR "Postpartum Hemorrhage/etiology"[Majr] ))) OR ("Pregnancy Complications"[Majr])) AND ((meta-analysis[Filter] OR review[Filter] OR systematicreview[Filter]) AND (fft[Filter]) AND (humans[Filter]) AND (female[Filter]) AND (data[Filter]) AND (english[Filter])))** Filters: **Full text, Associated data, Meta-Analysis, Review, English, Humans** | 2,230 | 04:07:00 |
| #33 |  |  | Search: **("Prevalence"[Mesh] OR "Epidemiology"[Mesh] OR "epidemiology" [Subheading]) AND ((((("Postpartum Hemorrhage"[Mesh]) OR (( "Postpartum Hemorrhage/epidemiology"[Majr] OR "Postpartum Hemorrhage/etiology"[Majr] ))) OR ("Pregnancy Complications"[Majr])) AND ((meta-analysis[Filter] OR review[Filter] OR systematicreview[Filter]) AND (fft[Filter]) AND (humans[Filter]) AND (female[Filter]) AND (data[Filter]) AND (english[Filter])))** Filters: **Full text, Associated data, Meta-Analysis, Review, Humans** | 2,230 | 04:06:52 |
| #32 |  |  | Search: **("Prevalence"[Mesh] OR "Epidemiology"[Mesh] OR "epidemiology" [Subheading]) AND ((((("Postpartum Hemorrhage"[Mesh]) OR (( "Postpartum Hemorrhage/epidemiology"[Majr] OR "Postpartum Hemorrhage/etiology"[Majr] ))) OR ("Pregnancy Complications"[Majr])) AND ((meta-analysis[Filter] OR review[Filter] OR systematicreview[Filter]) AND (fft[Filter]) AND (humans[Filter]) AND (female[Filter]) AND (data[Filter]) AND (english[Filter])))** Filters: **Full text, Associated data, Meta-Analysis, Review** | 2,230 | 04:06:47 |
| #31 |  |  | Search: **("Prevalence"[Mesh] OR "Epidemiology"[Mesh] OR "epidemiology" [Subheading]) AND ((((("Postpartum Hemorrhage"[Mesh]) OR (( "Postpartum Hemorrhage/epidemiology"[Majr] OR "Postpartum Hemorrhage/etiology"[Majr] ))) OR ("Pregnancy Complications"[Majr])) AND ((meta-analysis[Filter] OR review[Filter] OR systematicreview[Filter]) AND (fft[Filter]) AND (humans[Filter]) AND (female[Filter]) AND (data[Filter]) AND (english[Filter])))** Filters: **Full text, Associated data, Meta-Analysis** | 730 | 04:06:42 |
| #30 |  |  | Search: **("Prevalence"[Mesh] OR "Epidemiology"[Mesh] OR "epidemiology" [Subheading]) AND ((((("Postpartum Hemorrhage"[Mesh]) OR (( "Postpartum Hemorrhage/epidemiology"[Majr] OR "Postpartum Hemorrhage/etiology"[Majr] ))) OR ("Pregnancy Complications"[Majr])) AND ((meta-analysis[Filter] OR review[Filter] OR systematicreview[Filter]) AND (fft[Filter]) AND (humans[Filter]) AND (female[Filter]) AND (data[Filter]) AND (english[Filter])))** Filters: **Full text, Associated data** | 2,230 | 04:06:35 |
| #29 |  |  | Search: **("Prevalence"[Mesh] OR "Epidemiology"[Mesh] OR "epidemiology" [Subheading]) AND ((((("Postpartum Hemorrhage"[Mesh]) OR (( "Postpartum Hemorrhage/epidemiology"[Majr] OR "Postpartum Hemorrhage/etiology"[Majr] ))) OR ("Pregnancy Complications"[Majr])) AND ((meta-analysis[Filter] OR review[Filter] OR systematicreview[Filter]) AND (fft[Filter]) AND (humans[Filter]) AND (female[Filter]) AND (data[Filter]) AND (english[Filter])))** Filters: **Full text** | 2,230 | 04:06:29 |
| #28 |  |  | Search: **("Prevalence"[Mesh] OR "Epidemiology"[Mesh] OR "epidemiology" [Subheading]) AND ((((("Postpartum Hemorrhage"[Mesh]) OR (( "Postpartum Hemorrhage/epidemiology"[Majr] OR "Postpartum Hemorrhage/etiology"[Majr] ))) OR ("Pregnancy Complications"[Majr])) AND ((meta-analysis[Filter] OR review[Filter] OR systematicreview[Filter]) AND (fft[Filter]) AND (humans[Filter]) AND (female[Filter]) AND (data[Filter]) AND (english[Filter])))** | 2,230 | 04:06:00 |
| #27 |  |  | Search: **determinant** | 13,203,710 | 04:05:05 |
| #26 |  |  | Search: **magnitude** | 321,464 | 04:04:43 |
| #25 |  |  | Search: **prevalence** | 3,980,456 | 04:04:29 |
| #24 |  |  | Search: **"Prevalence"[Mesh] OR "Epidemiology"[Mesh] OR "epidemiology" [Subheading]** Sort by: **Most Recent** | 2,973,633 | 03:59:05 |
| #23 |  |  | Search: **(((("Postpartum Hemorrhage"[Mesh]) OR (( "Postpartum Hemorrhage/epidemiology"[Majr] OR "Postpartum Hemorrhage/etiology"[Majr] ))) OR ("Pregnancy Complications"[Majr]))** Filters: **Full text, Associated data, Meta-Analysis, Review, Systematic Review, English, Humans, Female** | 8,012 | 03:56:11 |
| #22 |  |  | Search: **(((("Postpartum Hemorrhage"[Mesh]) OR (( "Postpartum Hemorrhage/epidemiology"[Majr] OR "Postpartum Hemorrhage/etiology"[Majr] ))) OR ("Pregnancy Complications"[Majr]))** Filters: **Full text, Associated data, Meta-Analysis, Review, Systematic Review, English, Humans** | 8,256 | 03:56:04 |
| #21 |  |  | Search: **(((("Postpartum Hemorrhage"[Mesh]) OR (( "Postpartum Hemorrhage/epidemiology"[Majr] OR "Postpartum Hemorrhage/etiology"[Majr] ))) OR ("Pregnancy Complications"[Majr]))** Filters: **Full text, Associated data, Meta-Analysis, Review, Systematic Review, English** | 8,368 | 03:55:56 |
| #20 |  |  | Search: **(((("Postpartum Hemorrhage"[Mesh]) OR (( "Postpartum Hemorrhage/epidemiology"[Majr] OR "Postpartum Hemorrhage/etiology"[Majr] ))) OR ("Pregnancy Complications"[Majr]))** Filters: **Full text, Associated data, Meta-Analysis, Review, Systematic Review** | 8,716 | 03:55:12 |
| #19 |  |  | Search: **(((("Postpartum Hemorrhage"[Mesh]) OR (( "Postpartum Hemorrhage/epidemiology"[Majr] OR "Postpartum Hemorrhage/etiology"[Majr] ))) OR ("Pregnancy Complications"[Majr]))** Filters: **Full text, Associated data, Review, Systematic Review** | 8,424 | 03:55:03 |
| #18 |  |  | Search: **(((("Postpartum Hemorrhage"[Mesh]) OR (( "Postpartum Hemorrhage/epidemiology"[Majr] OR "Postpartum Hemorrhage/etiology"[Majr] ))) OR ("Pregnancy Complications"[Majr]))** Filters: **Full text, Associated data, Systematic Review** | 2,165 | 03:54:58 |
| #17 |  |  | Search: **(((("Postpartum Hemorrhage"[Mesh]) OR (( "Postpartum Hemorrhage/epidemiology"[Majr] OR "Postpartum Hemorrhage/etiology"[Majr] ))) OR ("Pregnancy Complications"[Majr]))** Filters: **Full text, Associated data** | 57,844 | 03:54:48 |
| #16 |  |  | Search: **(((("Postpartum Hemorrhage"[Mesh]) OR (( "Postpartum Hemorrhage/epidemiology"[Majr] OR "Postpartum Hemorrhage/etiology"[Majr] ))) OR ("Pregnancy Complications"[Majr])) AND ("Systematic Review" [Publication Type] AND "Systematic Reviews as Topic"[Mesh])) OR ("Meta-Analysis" [Publication Type] OR "Meta-Analysis as Topic"[Mesh] OR "Network Meta-Analysis as Topic"[Mesh] OR "Network Meta-Analysis" [Publication Type])** Filters: **Full text, Associated data** | 67,912 | 03:54:13 |
| #15 |  |  | Search: **(((("Postpartum Hemorrhage"[Mesh]) OR (( "Postpartum Hemorrhage/epidemiology"[Majr] OR "Postpartum Hemorrhage/etiology"[Majr] ))) OR ("Pregnancy Complications"[Majr])) AND ("Systematic Review" [Publication Type] OR "Systematic Reviews as Topic"[Mesh])) OR ("Meta-Analysis" [Publication Type] OR "Meta-Analysis as Topic"[Mesh] OR "Network Meta-Analysis as Topic"[Mesh] OR "Network Meta-Analysis" [Publication Type])** Filters: **Full text, Associated data** | 68,851 | 03:53:25 |
| #14 |  |  | Search: **(((("Postpartum Hemorrhage"[Mesh]) OR (( "Postpartum Hemorrhage/epidemiology"[Majr] OR "Postpartum Hemorrhage/etiology"[Majr] ))) OR ("Pregnancy Complications"[Majr])) AND ("Systematic Review" [Publication Type] OR "Systematic Reviews as Topic"[Mesh])) OR ("Meta-Analysis" [Publication Type] OR "Meta-Analysis as Topic"[Mesh] OR "Network Meta-Analysis as Topic"[Mesh] OR "Network Meta-Analysis" [Publication Type])** Filters: **Full text** | 238,826 | 03:53:15 |
| #13 |  |  | Search: **(((("Postpartum Hemorrhage"[Mesh]) OR (( "Postpartum Hemorrhage/epidemiology"[Majr] OR "Postpartum Hemorrhage/etiology"[Majr] ))) OR ("Pregnancy Complications"[Majr])) AND ("Systematic Review" [Publication Type] OR "Systematic Reviews as Topic"[Mesh])) OR ("Meta-Analysis" [Publication Type] OR "Meta-Analysis as Topic"[Mesh] OR "Network Meta-Analysis as Topic"[Mesh] OR "Network Meta-Analysis" [Publication Type])** | 246,527 | 03:52:39 |
| #12 |  |  | Search: **"Meta-Analysis" [Publication Type] OR "Meta-Analysis as Topic"[Mesh] OR "Network Meta-Analysis as Topic"[Mesh] OR "Network Meta-Analysis" [Publication Type]** Sort by: **Most Recent** | 243,110 | 03:50:46 |
| #11 |  |  | Search: **"Systematic Review" [Publication Type] OR "Systematic Reviews as Topic"[Mesh]** Sort by: **Most Recent** | 305,853 | 03:49:50 |
| #10 |  |  | Search: **"Pregnancy Complications"[Majr]** Sort by: **Most Recent** | 403,260 | 03:47:49 |
| #9 |  |  | Search: **( "Postpartum Hemorrhage/epidemiology"[Majr] OR "Postpartum Hemorrhage/etiology"[Majr] )** Sort by: **Most Recent** | 2,015 | 03:45:49 |
| #8 |  |  | Search: **"Postpartum Hemorrhage"[Mesh]** Sort by: **Most Recent** | 9,195 | 03:41:59 |
| #7 |  |  | Search: **Postpartum Hemorrhage : A Systematic Review and Meta-Analysis** Sort by: **Most Recent** | 417 | 03:37:53 |
| #6 |  |  | Search: **Similar articles for PMID: 35264188** Filters: **Full text, Meta-Analysis, Review, Systematic Review** | 579 | 03:35:24 |
| #5 |  |  | Search: **Similar articles for PMID: 35264188** Filters: **Full text, Review, Systematic Review** | 574 | 03:35:15 |
| #4 |  |  | Search: **Similar articles for PMID: 35264188** Filters: **Full text, Systematic Review** | 556 | 03:35:06 |
| #3 |  |  | Search: **Similar articles for PMID: 35264188** Filters: **Full text** | 725 | 03:34:59 |
| #2 |  |  | Search: **Similar articles for PMID: 35264188** | 730 | 03:34:49 |
| #1 |  |  | Search: **Magnitude and Determinants of Postpartum Hemorrhage in Sub-Saharan Africa: A Systematic Review and Meta-Analysis** Sort by: **Most Recent** | 4 | 03:20:13 |
